# Supplementary material for: Pressure and bias dependence of the rate-limiting steps of the oxygen reduction reaction
Source: Nat Commun. 2025 Dec 13;16:11170. doi: 10.1038/s41467-025-67494-x (PMC12708627; doi:10.1038/s41467-025-67494-x)
Supplement: Supplementary file 1 — Supplementary Information [file 41467_2025_67494_MOESM1_ESM.pdf]

Supplementary Information

Pressure and Bias Dependence of the Rate-Limiting Steps of the Oxygen Reduction Reaction

Alex Ricardo Silva Olaya[1‡], Jody Druce[1‡], Jose M. Gisbert-Gonzalez[1], Eduardo Ortega[1], Beatriz Roldan Cuenya[1], Sebastian Z. Oener[1*]

[1]*Department of Interface Science, Fritz-Haber Institute of the Max Planck Society, Berlin, Germany*

‡ These authors contributed equally.

*Corresponding author: oener@fhi-berlin.mpg.de

The PDF file includes:

Supplementary Notes 1-5

Supplementary Figures 1-32

Supplementary Tables 1-2

Supplementary References

Supplementary Note 1

Mass transport of membrane electrode assembly. The mass transport provided by gas diffusion electrodes (GDEs) in membrane electrode assemblies (MEA) reduces the possibility of pronounced temperature dependent mass transport limitations that could superimpose themselves onto the kinetic data in a manner to exactly produce Arrhenius linear fits with very high R^2 values (reaching 0.999 for Rh). In fact, Lazaridis et al.[1] discuss how MEAs can be used reliably to study catalyst kinetics, because they provide far superior mass transport over rotating disk electrodes (RDEs). Conversely, MEAs can access potential and current ranges that are inaccessible to RDEs, albeit at the complication of having to use nanoparticle catalysts inside ionomer dispersions in GDEs, instead of single crystal surfaces. As shown in Fig. 1d-f and shown in Supplementary Figures 20 and 24, this requires studies as function of catalyst loading to identify a low loading that reduces non-kinetic effects inside the GDEs, and where the rates scale with the loading (e.g. Fig. 1d-f). Next, we consider two possible limitations in detail, the H^+ conduction and O_2 transport. For water transport, we refer to Supplementary Note 2.

The H^+ transport in H_2-pump cells (Pt/c-Nafion-Pt/c) that use essentially the same half-cell than our study is far superior to any RDE experiments ever performed. Durst et al.[2], Sheng et al.[3] and Gisbert-Gonzalez et al.[4] showed that H_2-pump cells can be used to obtain almost textbook-like Butler-Volmer kinetics ($\alpha = \alpha_H = 0.5, \alpha_S = 0$) for very fast acidic HER on Pt group metals with exchange current densities exceeding values ever reported in RDEs[1]. Further, *Sheng et al.*[3] concluded that RDEs cannot overcome H^+ diffusional mass transport in acid to reliable study fast PGM HER kinetics. The excellent proton transport is also supported by Supplementary Figure 1 and very high current densities in a Pt/c-Nafion-Pt/c H_2-pump.

The O_2 transport in fuel cells is also clear better than in RDEs. For example, Gómez-Marín et al.[5] show how RDEs are eventually always impacted by the limited O_2 solubility for potentials < ~ 0.9 V_{RHE} in liquid electrolyte, almost independent of spin-speed. This can be circumvented in MEAs for a larger potential range. In Supplementary Figure 13, we show the rate constant of Fig. 2f in the main up to higher overpotentials. The mass transport limitation under these conditions only impact the kinetics more substantially for overpotentials > 425mV and absolute potentials <0.7V_{RHE}. For Ir/C and Rh/C the current densities are substantially lower and the kinetic regime is thus extended to much larger overpotentials.

Finally, we intentionally limited the mass transport by reducing the O_2-concentration in the O_2-feed to 20% by intermixing with Ar. The O_2 partial pressure is held constant to control the thermodynamics and equilibrium potential. Supplementary Figure 14a shows the temperature dependent polarization curves of a Pt/C fuel cell with 100% O_2 in the gas feed and Supplementary Figure 14b the exact same cell, but with substantially reduced O_2 concentration. Clearly, at higher overpotentials, insufficient O_2 mass transport reduces the currents. Supplementary Figure 14c shows the bias dependent changes in the kinetic maps that are almost identical at low overpotentials in the compensation region. However, for the O_2-poor conditions, the turning point occurs at slightly higher overpotentials and the absolute pre-exponential factor and activation before the turning point are slightly increased. However, after the turning point, the mass transport limitation might result in the substantial reduction of the pre-exponential factor. These results clearly show that the two kinetic regimes and the bias dependent Tafel slopes and charge transfer coefficients are not caused by mass-transport limitations, but can of course be influenced by it. In fact, our data indicates that mass transport limitations in liquid electrolytes can explain the single compensation region without a turning potential that has been observed previously in the literature using RDEs[6,7] and our own study[8]. Finally, we reiterate, that the turning potential coincides exactly with the maximum of the reduction wave on the Pt surface in absence of O_2, as shown in Fig. 1 of the main. This cannot be explained by a mass transport limitation.

Supplementary Note 2

Impact of water transport on GDE performance. The gas access to the catalyst nanoparticles is governed by gas and water transport through the gas diffusion electrode (GDE). During the oxygen reduction reaction (ORR), water is generated depending on the current density. In general, excess water can flood GDEs, which are conditioned under specific reaction conditions (e.g., -100 mA cm^{-2} at 60 °C). While flooding may not significantly impair performance when catalyst loading is high due to abundance of accessible sites, it becomes a critical factor at lower loadings. Collectively, our findings, however, suggest that the impact of GDE flooding is negligible at moderate to high catalyst loadings (e.g., 190 µg cm^{-2} and 551 µg cm^{-2}) and the current densities considered (~ 250 mA cm^{-2} for the in-depth kinetic analysis in Fig. 2-4):

1. Changing the temperature would have a larger impact on the water transport due to a large impact of the temperature on the water (vapor) saturation pressure. However, we observe very high linear regression values reaching 0.99 in the Arrhenius analysis of the temperature dependent rates. A temperature dependent water concentration in the GDE would lead to pronounced temperature dependent Arrhenius curves.

2. We observe that the linear regression values for the lowest loading (i.e. ~20 µg cm^{-2}) tend to be lower, which might indicate an impact of non-ideal water transport and a larger susceptibility of flooding in the GDE with a lower number of active sites. For medium and high loadings, we observe large R^2 values, as detailed above.

3. Flooding would impact the kinetic analysis increasingly with increasing current density, as more water is generated and more O_2 needs to be consumed to maintain the currents. However, our R^2 values tend to increase with current density, not decrease.

4. We limit the in-depth kinetic analysis to current densities of ~ 250 mA cm^{-2} in Fig. 2-4. In Fig. 1, we show the kinetics and analysis to higher current densities, where mass transport increasingly impacts the polarization curves and extracted kinetics. This can be seen by the constant differential resistance in the polarization curves.

5. The bias dependent activation volume, $\Delta V_\eta^\ddagger$, extracted from the pressure dependent current densities (Supplementary Note 4) at fixed temperatures shows very similar relative changes as the bias dependent activation energy, E_A^η, and Arrhenius pre-exponential factor, $\log A^\eta$, that were extracted from the temperature dependent current densities at a fixed pressure (Figure 1). In other words, if a pressure-dependent mass transport limitation would be the origin of activation volume, $\Delta V_\eta^\ddagger$, these changes should not appear at a constant pressure for E_A^η and $\log A^\eta$. This holds for the current density range ≤ 250 mA cm^{-2}, which shows well-behaved polarization curves with a decreasing differential resistance (see e.g. Fig. 2a).

Supplementary Note 3

Real and formal activation parameters. The activation energy and pre-exponential factor can be obtained *via* Arrhenius analysis of temperature dependent currents as function of overpotential, η, or absolute electrode potential, E_{RHE}.[10] The *real* activation energy and pre-exponential factor as a function of overpotential are defined as

$$E_A^{\eta} = -R\left(\frac{\partial lnj(\eta)}{\partial T^{-1}}\right)_{\eta} \quad 3$$

$$\ln A^{\eta} = lnj(\eta)_{T\to\infty} \quad 4$$

The *formal* activation energy and pre-exponential factor as a function of absolute potential are defined as

$$E_A^{RHE} = -R\left(\frac{\partial lnj(\eta)}{\partial T^{-1}}\right)_{\eta} \quad 5$$

$$\ln A^{RHE} = lnj(E_{RHE})_{T\to\infty} \quad 6$$

For this study, the difference between the real and formal activation parameters arises due to the temperature and pressure dependence of the equilibrium potential of the ORR (see Methods). Experimentally, one collects a set of temperature dependent polarization curves at defined potentials referenced vs. a fixed absolute electrode (SHE or RHE). This absolute potential can be related to surface processes, such as to a reduction peak on the SHE/RHE scale. When converting to the overpotential scale, η, one needs to consider the temperature (and pressure) dependence of the equilibrium potential, $E^0(T,P)$. Conversely, the temperature and pressure dependent currents at a fixed overpotential value are extracted from currents across a range of absolute potentials vs. SHE. This complicates to link a surface process on the SHE or RHE scale to the real activation parameters. The activation energy and pre-exponential factor at a fixed overpotential are extracted from currents across a range of potentials on the RHE scale. On the other hand, the real activation parameters are important, because they directly inform on the impact of the applied electrochemical potential gradient and, thus, free energy driving force, on the kinetics. This difference is highlighted in Figure 1 and Supplementary Figure 16.

Supplementary Note 4

Calculation of the reaction order with respect to oxygen. The oxygen reduction reaction in acidic media is described by the balanced equation:

$$O_2 + 4(H^+ + e^-) \rightarrow 2H_2O \quad (7)$$

The process involves four protons and four electrons per oxygen molecule and leads to the formation of water. While the ORR proceeds via multiple elementary steps, the overall current measured experimentally is related to the net rate, v_{ORR}. Therefore, a global rate law can be formulated, irrespective of the specific pathway, or rate-determining step:

$$v_{ORR} = \frac{j_{ORR}(\eta, T, [x])}{4F} = [O_2]^n [H^+]^m [H_2O]^o k_{ORR}(\eta, T) \quad (8)$$

with measured current density for ORR, j_{ORR}, the overpotential, η, the temperature, T, and Faraday's constant, F. The exponents n, m, and o represent the reaction orders with respect to oxygen, protons, and water, respectively.

Several simplifications can be made based on the experimental control variables:

First, the activity of water is effectively constant throughout the measurement due to the large excess of water present in the Nafion membrane and ionomer-catalyst layer (see also Supplementary Note 2). The water is needed to ensure high proton conductivity. Thus, we set the reaction order with respect to water (o) to zero.

Second, the proton activity is similarly stable and can be considered non-limiting, because the Nafion membrane maintains a stable low pH (~0) at the measured current densities. The Nafion concentration is very high, as also demonstrated in the hydrogen pump configuration (Supplementary Figure 1). 100mV are needed to drive current densities up to 1A cm^{-2}, which can be corrected for with regular ohmic drop correction. Finally, the HOR counter-electrode kinetics are very fast, supplying protons at a high rate to directly balance the ORR. Given these factors, the reaction order with respect to protons (m) can also be assumed to be zero.

In the main, we discuss the importance of cross-checking the results that are obtained from the temperature dependence of the currents at constant pressure (bias dependent E_A and logA) and pressure dependence at constant temperature (bias dependent $\Delta V^\ddagger$, see Supplementary Note 5). Further, when we use the pressure dependence to isolate the bias dependent k_{ORR} and isolate its temperature dependence we obtain a bias dependent kinetic map (Supplementary Figure 20f) that is very similar to the one obtained purely from the temperature dependence at constant pressures (Figure 1d and 2b). Such cross-verification across the bias dependent activation parameters can exclude any substantial impact of pressure or temperature dependent changes in the water or proton concentration and, thus, allows us to set the respective reaction order to zero in this study. We note, if we were able to obtain control over the proton concentration inside the MEA, we might also have to consider a proton reaction order that is non-zero and, thus, would have to expand the rate equation. Our approach rests on varying experimental control parameters to observe apparent changes in the bias dependent activation parameters.

Conversely, we simplify the rate law x to:

$$v_{ORR} = k_{ORR}(\eta, T)[O_2]^n \quad (9)$$

We relate the concentration of O_2, $[O_2]$, to its partial pressure, pO_2, assuming ideal gas behavior at the triple-phase boundary over the used temperature and pressure range, i.e. $[O_2] = pO_2/RT$.

Substituting into the rate expression gives:

$$v_{\mathrm{ORR}} = \frac{j_{\mathrm{ORR}}(\eta, T, [p\mathrm{O_2}])}{4\mathrm{F}} = k_{\mathrm{ORR}}(\eta, T)\left[\frac{p\mathrm{O_2}}{\mathrm{R}T}\right]^n \qquad 10$$

This expression can be linearized by taking the natural logarithm:

$$\ln j_{\mathrm{ORR}}(\eta, T, [p\mathrm{O_2}]) = \ln k_{\mathrm{ORR}}(\eta, T) + \ln 4\mathrm{F} + \mathrm{n}\ln\left[\frac{p\mathrm{O_2}}{\mathrm{R}T}\right] \qquad 11$$

Or in base-10 form:

$$\log_{10} j_{\mathrm{ORR}}(\eta, T, [p\mathrm{O_2}]) = \log_{10} k_{\mathrm{ORR}}(\eta, T) + \log_{10} 4\mathrm{F} + \mathrm{n}\log_{10}\left[\frac{p\mathrm{O_2}}{\mathrm{R}T}\right] \qquad 12$$

Thus, by plotting $\log_{10} j_{\mathrm{ORR}}$ versus $\log_{10}(p\mathrm{O_2}/\mathrm{R}T)$ at constant overpotential and temperature, the reaction order *n* with respect to oxygen can be obtained directly from the slope.

To evaluate whether the rate constant k_{ORR} depends on pressure, the derivative of the logarithmic rate expression with respect to the oxygen pressure can be taken:

$$\frac{\partial \ln j_{\mathrm{ORR}}(\eta, T, [p\mathrm{O_2}])}{\partial p\mathrm{O_2}} = \frac{\partial \ln k_{\mathrm{ORR}}(\eta, T)}{\partial p\mathrm{O_2}} + n\frac{1}{p\mathrm{O_2}} \qquad 13$$

Therefore, plotting $\Delta \ln j_{\mathrm{ORR}}/\Delta p\mathrm{O_2}$ versus $1/pO_2$ provides an independent means to determine the reaction order *n* from the slope, while the intercept reveals any pressure dependence in the rate constant k_{ORR}, which can be related to an apparent activation volume.

Supplementary Note 5

Derivation of $\Delta H^{\ddagger}$, $\Delta S^{\ddagger}$ and $\Delta V^{\ddagger}$. The empirical Arrhenius (eq. 14) equation finds its theoretical foundation in the Eyring (eq. 15) equation[11]. For the simplest case of ideal gasses in Boltzmann statistics, where molecules are considered to act as point particles interacting with perfectly elastic collisions, the probability ($P(E)$) that a mole of molecules has a certain energy (E) (eq. 16) is proportional to the exponential of the negative energy.

$$k = A\mathrm{e}^{\frac{-E_A}{\mathrm{R}T}} \quad 14$$

$$k = \frac{\mathrm{k_B}T}{\mathrm{h}}\mathrm{e}^{\frac{-\Delta G^{\ddagger}}{\mathrm{R}T}} \quad 15$$

$$P(E) = \frac{\mathrm{e}^{\frac{-E}{\mathrm{R}T}}}{Z} \quad 16$$

Where R is the universal gas constant, *T* is absolute temperature and *Z* is the partition function.

The complex intermolecular forces present in liquids, especially in aqueous systems, mean this simple exponential dependence may not necessarily have to hold for all the potential energy contributions[12]. However, the rates of many chemical reactions, diffusion, ionic conduction and other processes in liquids have been shown experimentally to have Arrhenius-like behavior[13–17]. Additionally, the entropic term described by the Eyring equation, believed to contribute to the pre-exponential factor, may account for the contributions of these complex intermolecular interactions to the rate, even without a rigorous description of the potential energy terms. Thus, the law is often a good description of liquid-phase kinetics, despite the non-Boltzmann nature of many intermolecular forces in liquids.

In order to describe processes analyzed with the Arrhenius equation within the Eyring framework, the product of the pre-factor and entropic term were equated to the pre-exponential factor, and the enthalpic term was considered to represent the activation energy term. Strictly, the enthalpy in the Eyring equation is equal to the activation energy plus the contribution of *PV* work. However, due to the relatively low compressibility of condensed phases, this term is considered negligible (and essentially constant in this temperature range) and consequently the enthalpy is only determined by the activation energy.

The entropy of activation ($\Delta S^{\ddagger}$) was calculated using the pre-exponential factor and the experimental temperature for a given potential and pressure (eq. 23). The units of the pre-exponential factor are in Amps, accounting for the surface area or loading. Similarly, the enthalpy of activation ($\Delta H^{\ddagger}$) was simply taken as the activation energy (eq. 20). Note, the transmission coefficient κ is assumed to be 1, as is typically done for adiabatic processes.

$$\ln k = \left(\frac{-E_A}{\mathrm{R}}\right)\left(\frac{1}{T}\right) + \ln A \quad 17$$

$$k = \frac{\kappa \mathrm{k_B}T}{\mathrm{h}}\mathrm{e}^{\left(\frac{\Delta S^{\ddagger}}{\mathrm{R}}\right)}\mathrm{e}^{\left(\frac{-\Delta H^{\ddagger}}{\mathrm{R}T}\right)} \quad 18$$

$$\ln k = \left(\frac{-\Delta H^{\ddagger}}{\mathrm{R}}\right)\left(\frac{1}{T}\right) + \frac{\Delta S^{\ddagger}}{\mathrm{R}} + \ln\left(\frac{\mathrm{k_B}T}{\mathrm{h}}\right) \quad 19$$

$$\Delta H^{\ddagger} \cong E_A \quad 20$$

$$\ln A = \frac{\Delta S^{\ddagger}}{\mathrm{R}} + \ln\left(\frac{\mathrm{k_B}T}{\mathrm{h}}\right) \qquad 21$$

$$\Delta S^{\ddagger} = \mathrm{R}\left[\ln A - \ln\left(\frac{\mathrm{k_B}T}{\mathrm{h}}\right)\right] \qquad 22$$

$$\Delta S^{\ddagger} = \mathrm{R}\ln\left(\frac{\mathrm{h}A}{\mathrm{k_B}T}\right) \qquad 23$$

Where A is the pre-exponential factor, E_A is the activation energy, $\mathrm{k_B}$ is the Boltzmann constant, h is Planck's constant and $\Delta G^{\ddagger}$ is the free energy of activation.

The volume of activation ($\Delta V^{\ddagger}$) for each potential was calculated at constant temperature using its definition in statistical mechanics (eq. 24). Because the potentials used across pressures were not identical, an evenly-spaced matrix was used to produce a consistent set of potentials, interpolating currents based on the experimental IV curves. $\Delta V^{\ddagger}$ was calculated from[18]

$$\Delta V^{\ddagger} = -\mathrm{R}T\left(\frac{\partial \ln k}{\partial P}\right)_T \qquad 24$$

The calculation of the three activation parameters (activation energy, activation entropy, and activation volume) relies on determining the reaction rate constant as a function of temperature and overpotential. In this study, the reaction rate was obtained from the measured current density associated with the ORR, as outlined in Supplementary Note 4. This approach enables the application of transition state theory and Arrhenius-type analyses to extract the kinetic parameters that govern the ORR general process.

The use of electrochemical surface area (ECSA) was complicated by the differences in best-practice calculation between Pt and Rh. If ECSA from the hydrogen underpotential deposition zone (HUPD) would have been extracted, it is very questionable whether this area would be relevant for oxygen rather than hydrogen adsorption. This is further exasperated by the potential dependent changes on the catalyst surface that are known to change the number of active sites. Additionally, normalization by mass would have disregarded the relatively large differences in surface area between the two investigated metals reported by the commercial provider (see Supplementary Table 1). In any case, the potential dependent changes in $\Delta H^{\ddagger}$, $\Delta S^{\ddagger}$ and $\Delta V^{\ddagger}$ are unaffected by the normalization procedure. This would only change the absolute value of log A and in extension the activation entropy, but not the relative changes. For the impact of the different normalization procedures on the kinetic maps, see Supplementary Figures 18, 22-23.

In this work, $\Delta H^{\ddagger}$, $\Delta S^{\ddagger}$ and $\Delta V^{\ddagger}$ are found to vary with the overpotential. The fact that all three are functions of bias suggests that the mechanism by which they are changed is common. We hypothesize that these relationships are the consequence of the direct dependence of all activation parameters on the transition state and reactant partition functions ($Z^{\ddagger}$ and Z_R respectively). These relations are derived in equations 25-33 below. More explicitly, we propose that the common partition function is overpotential-dependent.

$$\Delta G^{\ddagger} = \Delta H^{\ddagger} - T\Delta S^{\ddagger} \qquad 25$$

$$\Delta H^{\ddagger} = \Delta G^{\ddagger} - T\left(\frac{\partial \Delta G^{\ddagger}}{\partial T}\right)_P \qquad 26$$

$$\Delta G^{\ddagger} = -RT\left(\ln Z^{\ddagger} - \ln Z_R\right) \qquad 27$$

$$\left(\frac{\partial \Delta G^{\ddagger}}{\partial T}\right)_P = \frac{\partial}{\partial T}\left[-\mathrm{R}T\left(\ln Z^{\ddagger} - \ln Z_R\right)\right] \quad 28$$

$$\Delta H^{\ddagger} = \mathrm{R}T^2\left[-\frac{\partial}{\partial T}\left(\ln\left(Z^{\ddagger}\right)\right) + \frac{\partial}{\partial T}\left(\ln\left(Z_R\right)\right)\right] \quad 29$$

$$\Delta S^{\ddagger} = -\left[\frac{\partial \Delta G^{\ddagger}}{\partial T}\right]_P \quad 30$$

$$\Delta S^{\ddagger} = \mathrm{R}\left[\left(\ln Z^{\ddagger} - \ln Z_R\right) + T\left(\frac{\partial}{\partial T}\left(\ln Z^{\ddagger}\right) - \frac{\partial}{\partial T}\left(\ln Z_R\right)\right)\right] \quad 31$$

$$\Delta V^{\ddagger} = \left[\frac{\partial \Delta G^{\ddagger}}{\partial P}\right]_T \quad 32$$

$$\Delta V^{\ddagger} = -\mathrm{R}T\left[\frac{\partial}{\partial P}\left(\ln Z^{\ddagger}\right) - \frac{\partial}{\partial P}\left(\ln Z_R\right)\right] \quad 33$$

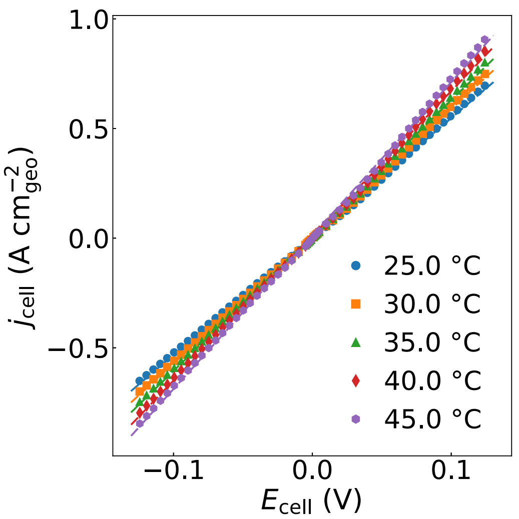

Supplementary Figure 1 | Determination of the overpotential for the hydrogen oxidation at the anode. Polarization curves were obtained by chronoamperometry at various applied potentials and temperatures using a symmetric cell configuration (H_2 | Pt@C | Nafion 212 | Pt@C | H_2), employing the same high loading Pt/C electrodes and 2bar H_2 pressure used in the MEA of the main ORR study. The resulting plots show a linear relationship between current and overpotential, indicating that the hydrogen oxidation reaction (HOR) overpotential can be reliably approximated as a linear function of the measured current. The HOR overpotential at a given current was estimated as half the potential extrapolated from this linear trend in the symmetric H_2 pump cell.

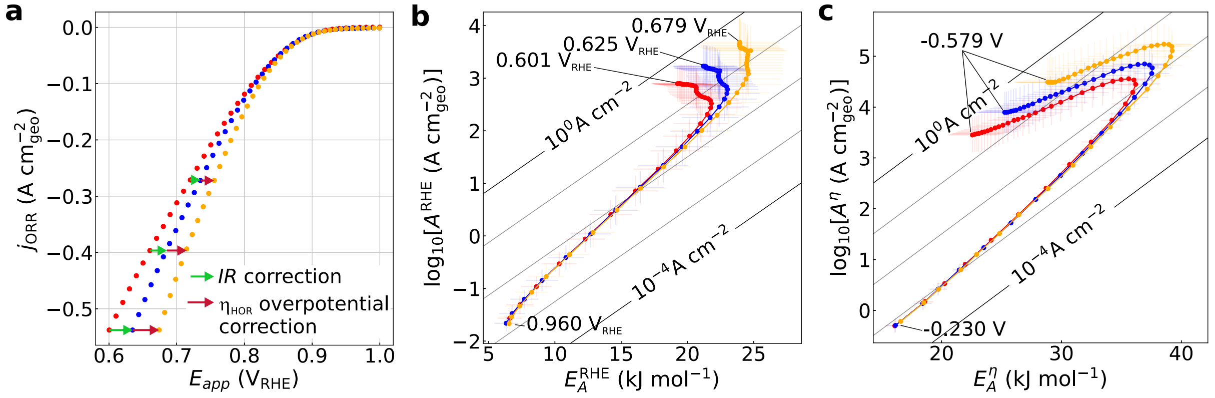

Supplementary Figure 2 | Corrections to the cell potential and their impact on ORR kinetics. a, ORR polarization curve measured at 45 °C (red). Subtracting the ohmic drop (*IR*) from the applied potential shifts the data points to higher potentials (green arrows), producing the corrected blue curve. A further correction is applied by subtracting the anode potential required for the hydrogen oxidation reaction (HOR), determined by interpolation from the polarization curves in Supplementary Figure 1. This results in an additional shift to higher absolute potentials (red arrows), yielding the final yellow curve. **b,** Kinetic maps derived from Arrhenius analysis of fully corrected temperature dependent current-potential curves using the polarization curves expressed on the RHE scale: uncorrected data (red), after IR correction (blue), and after both IR and HOR corrections (yellow). The two kinetic regimes are already distinguishable without corrections. Each correction primarily affects the high-potential region, with minimal impact on the compensation zone. **c,** Overpotential dependent kinetic maps for the same three correction stages: uncorrected (red), IR-corrected (blue), and fully corrected (yellow). Due to the temperature dependence of the equilibrium potential, the high-overpotential region differs from the RHE-scale map, as discussed in Supplementary Figure 14.

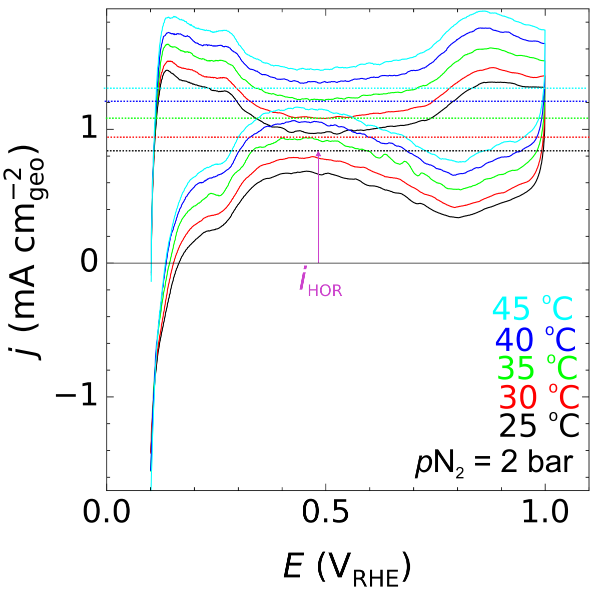

Supplementary Figure 3 | H_2 crossover through the Nafion membrane and its impact on the ORR current. A cyclic voltammetry was performed under N_2 atmosphere in the anode compartment at the same temperatures and pressures used in the main study to evaluate the electrochemical state of the Pt nanoparticles and to quantify the hydrogen oxidation current at the cathode, resulting from H_2 crossover through the Nafion membrane (~50 µm thick). This crossover current appears as a baseline offset in the voltammogram relative to zero current and is attributed to the electrochemical oxidation of permeated hydrogen. While this hydrogen oxidation current could influence the measured ORR current at very low overpotentials, its impact becomes negligible at higher overpotentials, where ORR currents reach >10 mA cm^{-2}.

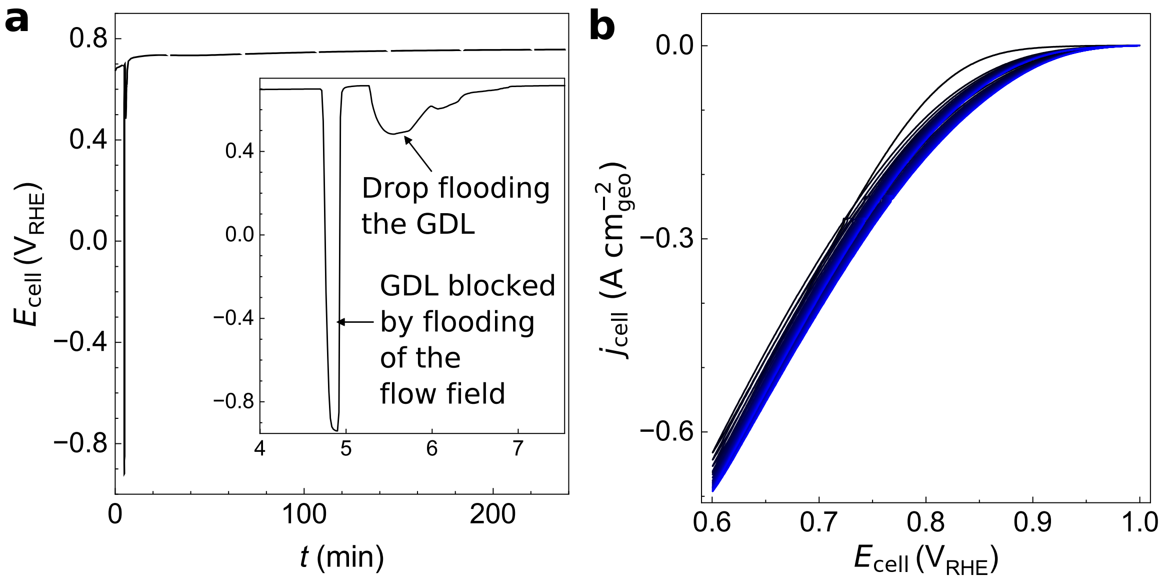

Supplementary Figure 4 | MEA cell conditioning and electrode stabilization. a. Chronopotentiometric response of the O_2|Pt@C|Nafion|Pt@C|H_2 cell during MEA conditioning at -100 mA cm^{-2}. The Pt loading for the ORR GDE was 0.19 mg cm^{-2}. The potential increased gradually over the first 120 minutes, with two peaks at the start (inset) indicating initial water management challenges. A stable potential was reached after ~80 minutes, rising to 0.757 V_{RHE} as temperature, humidity, and water management equilibrated. Gas pressure was maintained at 2 bar (abs). **b.** Cyclic voltammograms at 10 mV s^{-1} after initial steady-state conditioning. The cell underwent 180 cycles, stabilizing with negligible differences between cycles. Every 10th cycle is shown for clarity, transitioning from black (1st cycle) to dark blue (final cycles). Early cycles exhibited higher ORR onset potentials, with later cycles converging, indicating electrode activation and surface stabilization. Assembly involved embedding the HOR GDL electrode, positioning the humidified Nafion membrane, and securing the ORR electrode with a compression jacket, all at room temperature.

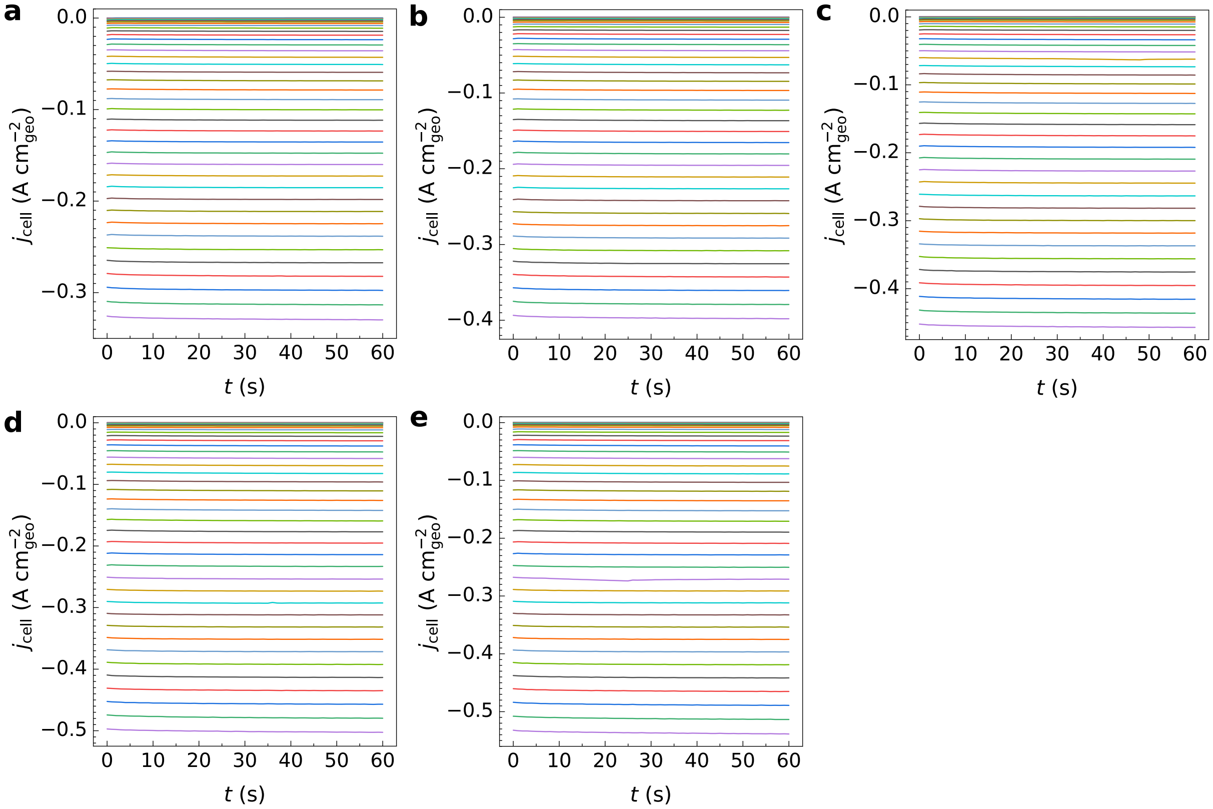

Supplementary Figure 5 | Chronoamperometries for constructing steady-state current-potential curves. Chronoamperometric measurements of the ORR on Pt nanoparticles (0.19 mg cm^{-2}) were conducted at **a,** 25 °C; **b,** 30 °C; **c,** 35 °C; **d,** 40 °C and **e,** 45 °C under pH_2 = 2 bar and pO_2 = 2 bar. To ensure steady-state conditions, chronoamperometries were performed at sequential potentials, starting from the low overpotential limit (upper RHE potential) and increasing by 10 mV steps to the high overpotential limit (lower RHE potential). The current was recorded for 60 s at each potential, and the average of the final 15 s was taken as the steady-state current for the imposed potential.

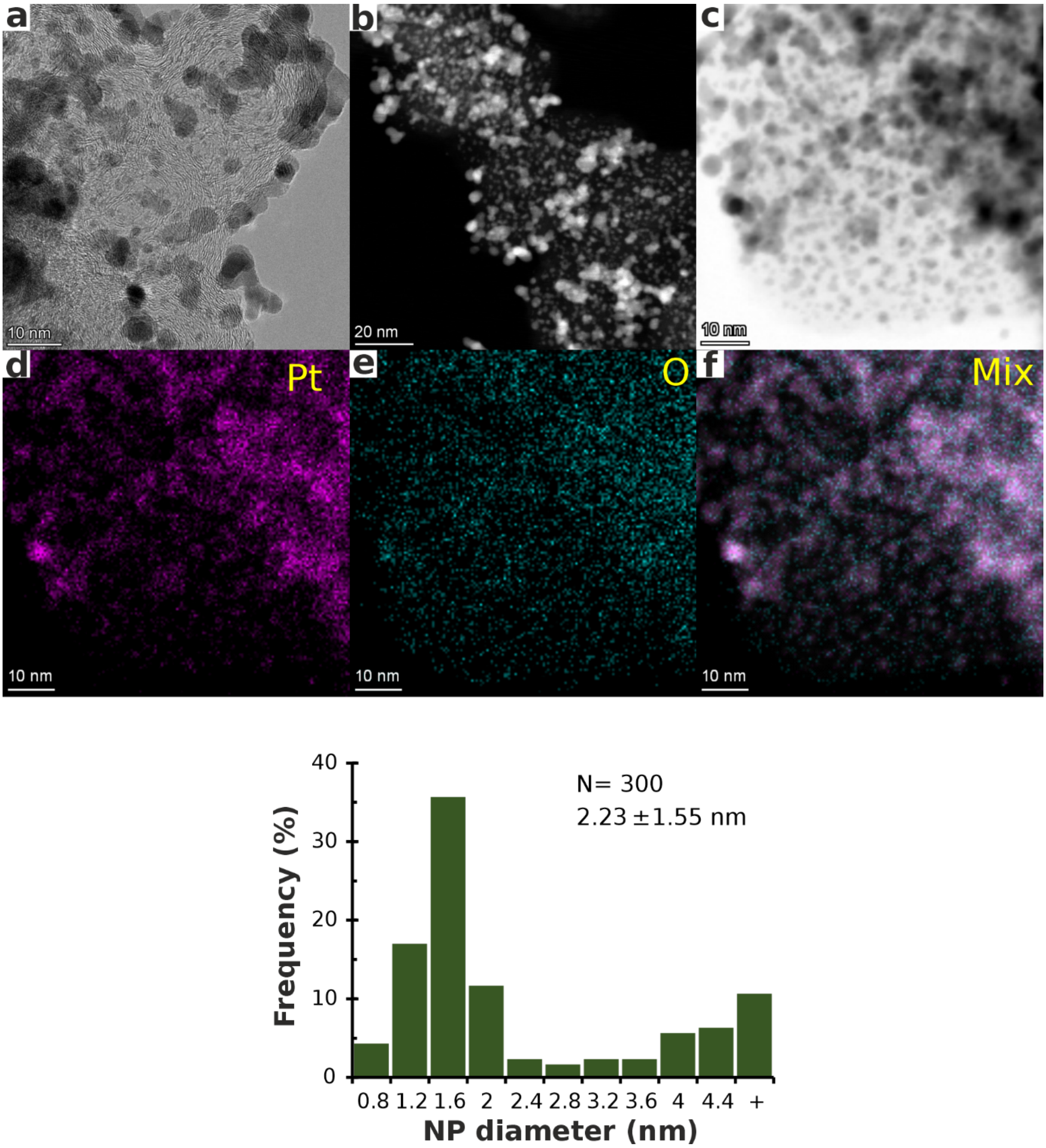

Supplementary Figure 6 | Characterization of Pt nanoparticles as received. a, TEM; **b,** Dark-Field and **c,** Bright-Field images of the Pt nanoparticles supported on the Ketjenblack carbon as received from the vendor. The heterogeneity of the particle size is apparent. **d-f,** Energy dispersive X-ray spectroscopy (EDX) indicates absence of Pt oxide. The histogram after evaluating 300 nanoparticles shows a size distribution with a mean particle diameter of 2.23 nm and a peak around 1.6 nm.

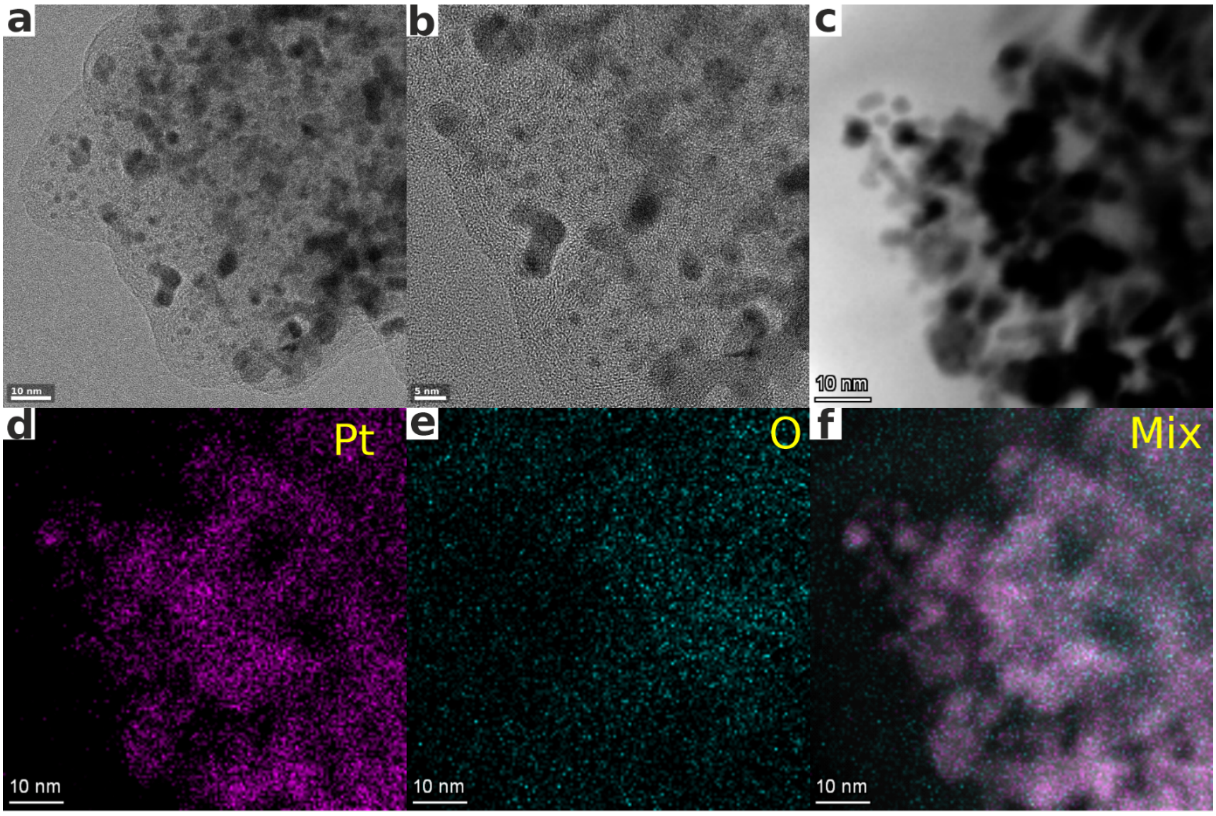

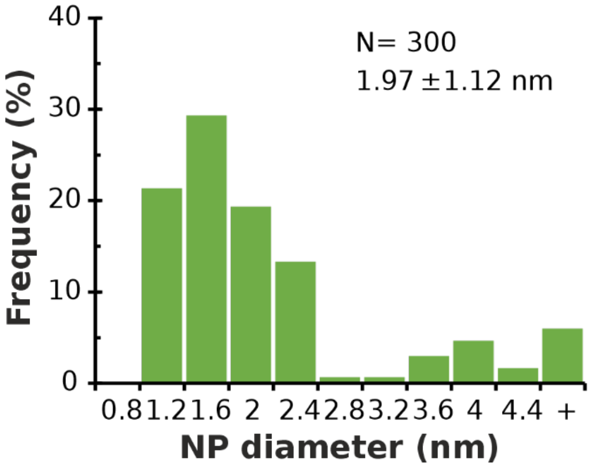

Supplementary Figure 7 | Characterization of Pt nanoparticles after the conditioning in the MEA. a-b, TEM and, **c,** Bright-Field images of the Pt nanoparticles after the preparation of the electrodes by spray coating and conditioning. The Bright-Field image was challenging to obtain due to the fast formation of contamination during acquisition, likely stemming from ionomer residues. **d-f,** EDX shows no presence of Pt oxide formation. The histogram obtained evaluating 300 nanoparticles shows a similar size distribution, as the received particles, but with a slightly lower particle size.

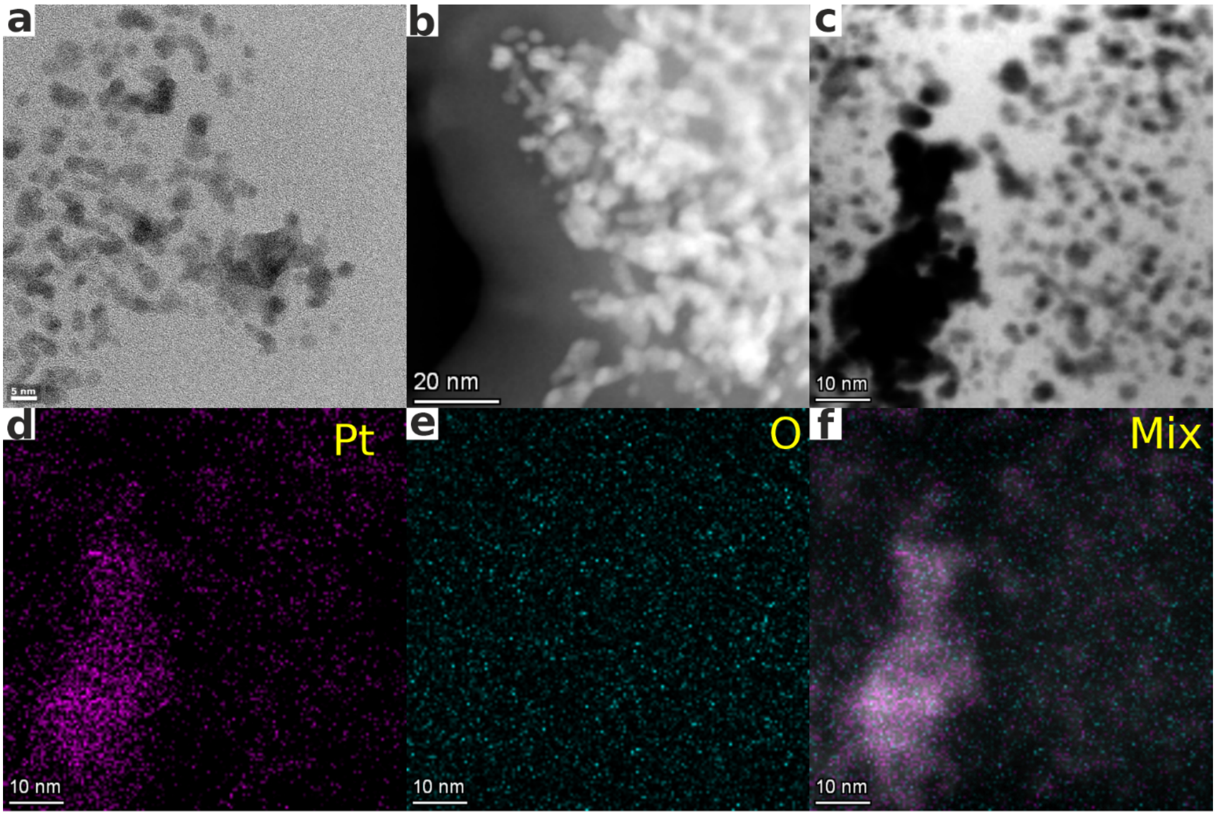

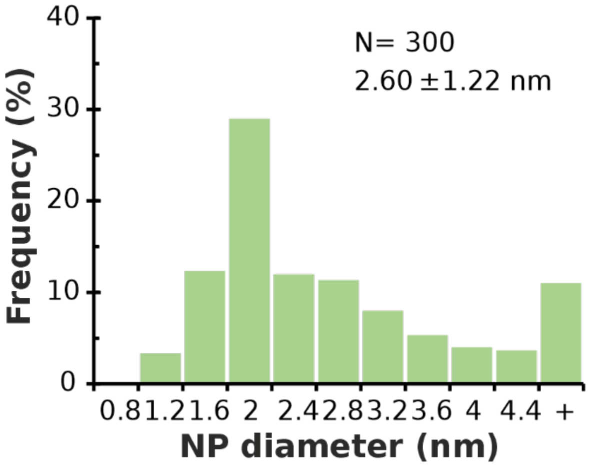

Supplementary Figure 8 | Characterization of Pt nanoparticles after extended ORR studies at different potentials, temperatures and oxygen pressures. a, TEM, **b,** Dark-Field and **c,** Bright-Field images of the Pt nanoparticles after the ORR (final state). The Dark and Bright-Field images were challenging to obtain due to the fast formation of contamination during the acquisition of the images, likely stemming from ionomer residues. **d-f,** EDX shows no presence of Pt oxide formation. The histogram obtained evaluating 300 nanoparticles shows a wider distribution of particle sizes compared with the histogram of the material before and after conditioning. However, the mean particle size is just slightly higher than the "as received" material.

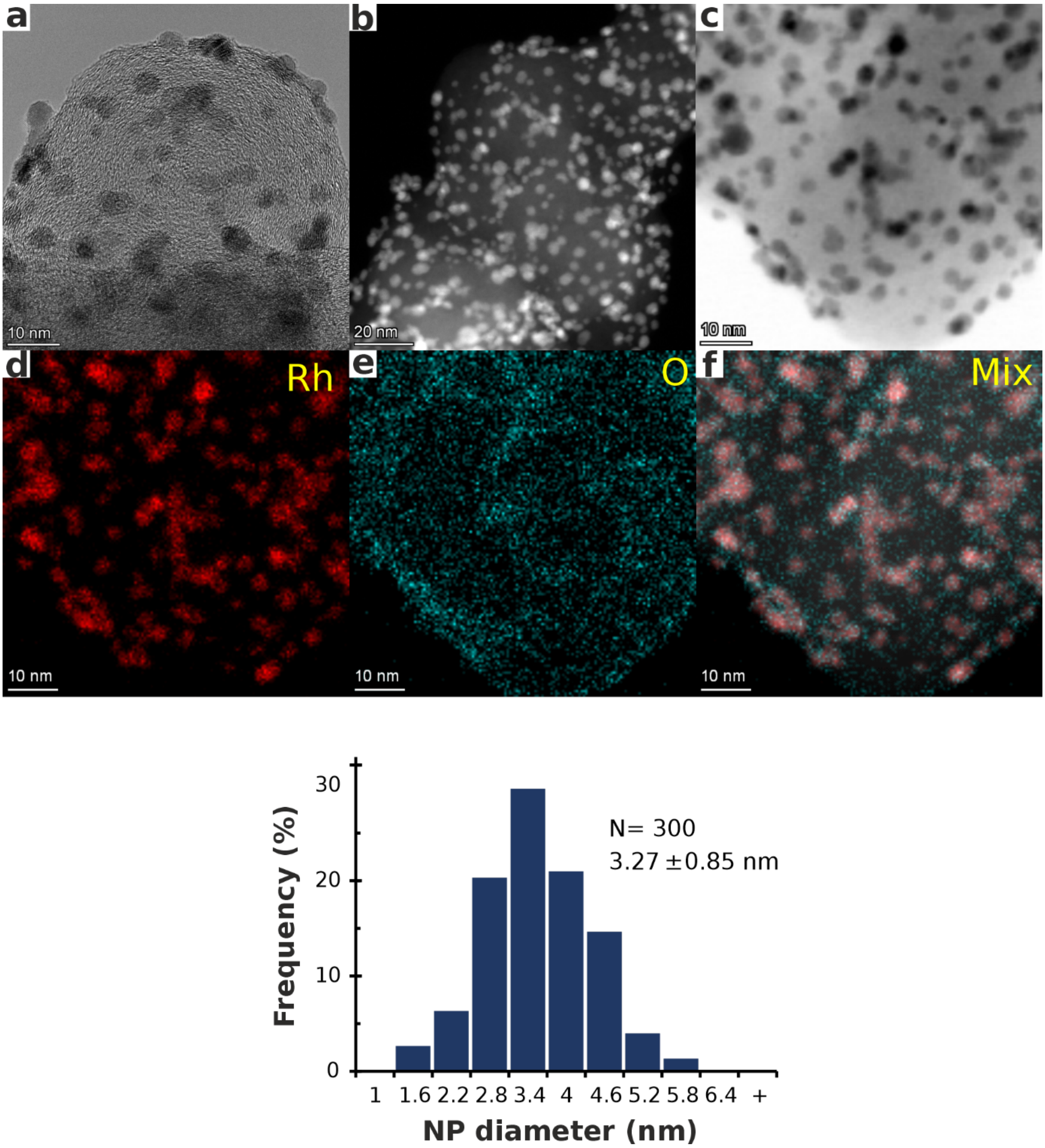

Supplementary Figure 9 | Characterization of Rh nanoparticles as received. a, TEM; **b,** Dark-Field and **c,** Bright-Field images of the Rh nanoparticles supported on the Vulcan carbon as received from the vendor. Compared to the Pt/C, individual nanoparticles can be resolved more clearly due to the lower metal loading on the carbon support (see Supplementary Table 1). **d-f,** EDX shows no clear presence of rhodium oxide. The histogram obtained evaluating 300 nanoparticles shows a normal distribution of the particle size with a mean particle diameter of 3.27 nm.

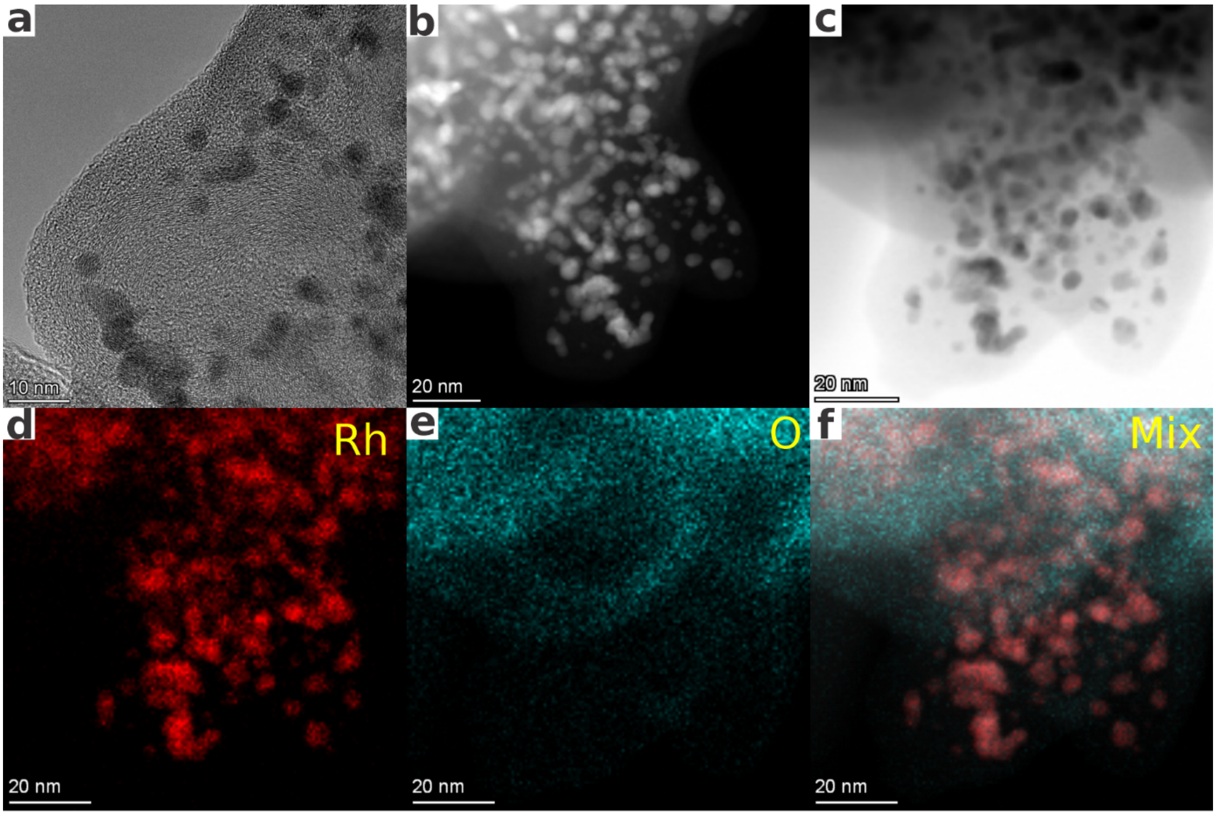

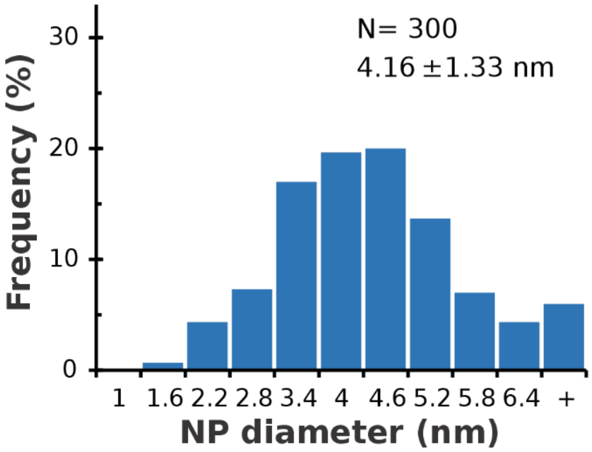

Supplementary Figure 10 | Characterization of Rh nanoparticles after conditioning in the MEA. a TEM **b,** Dark-Field and **c,** Bright-Field images of the Rh nanoparticles after the preparation of the electrodes by spray coating and the conditioning as indicated showed in the Supplementary Figure 4. **d-f,** EDX shows no presence of Rh oxide formation. The histogram obtained evaluating 300 nanoparticles shows a more disperse distribution compared with the "as received" material, with a slightly higher mean particle size.

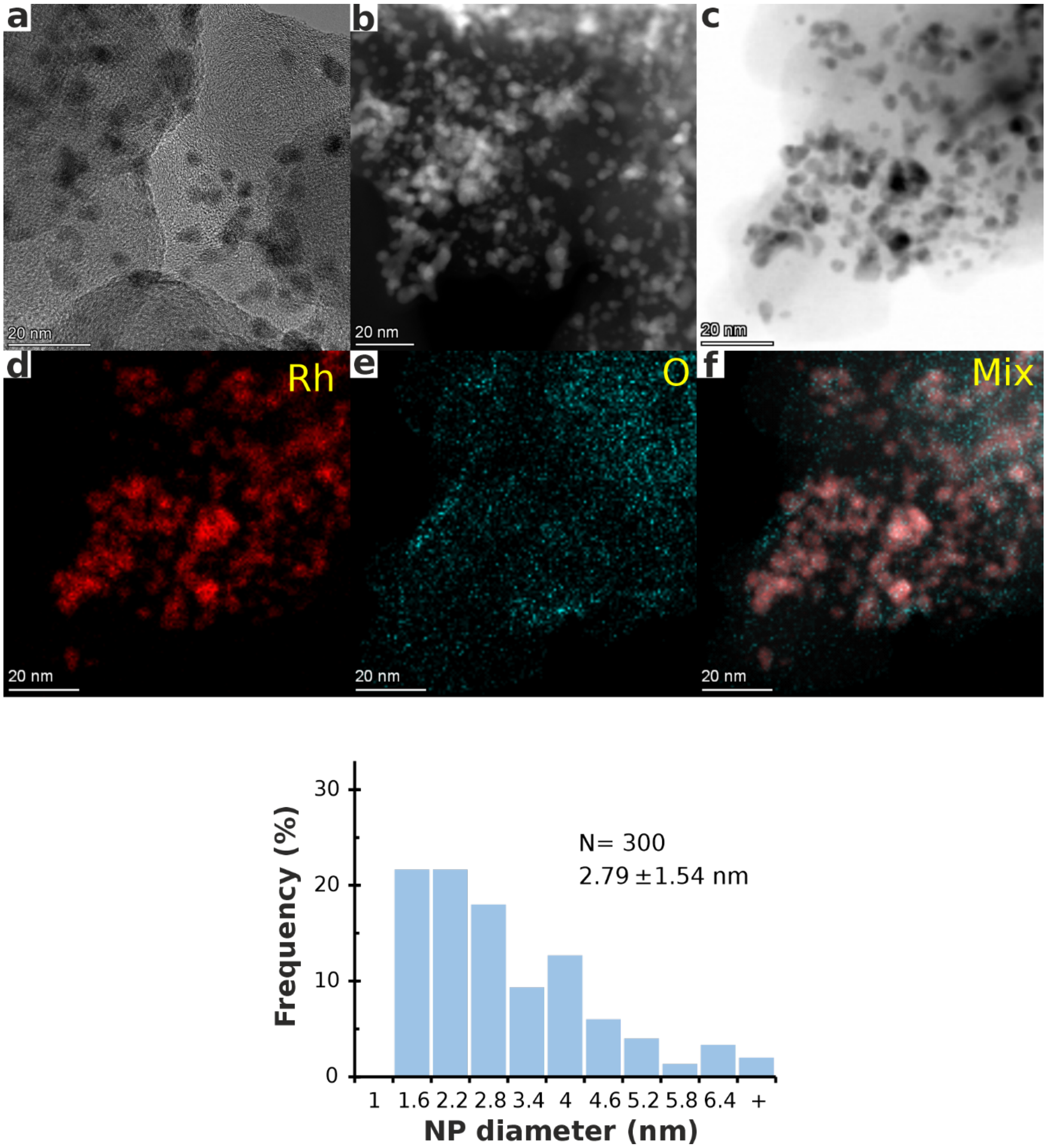

Supplementary Figure 11 | Characterization of Rh nanoparticles after extended ORR studies at different potentials, temperatures and oxygen pressures. a TEM **b,** Dark-Field and **c,** Bright-Field images of the Rh nanoparticles after the ORR experiments (final state). Well dispersed nanoparticles are still apparent after the experiments. **d-f,** EDX shows no presence of rhodium oxide formation. The histogram obtained evaluating 300 nanoparticles shows a shift of the particle size distribution to lower values compared with the histogram of the material after conditioning (mean ~ 4.1 nm). As shown in Supplementary Figure 9, the as-received samples have a mean particle size of ~ 3.27 nm. This suggests that Rh redistributes on the carbon support during the reaction.

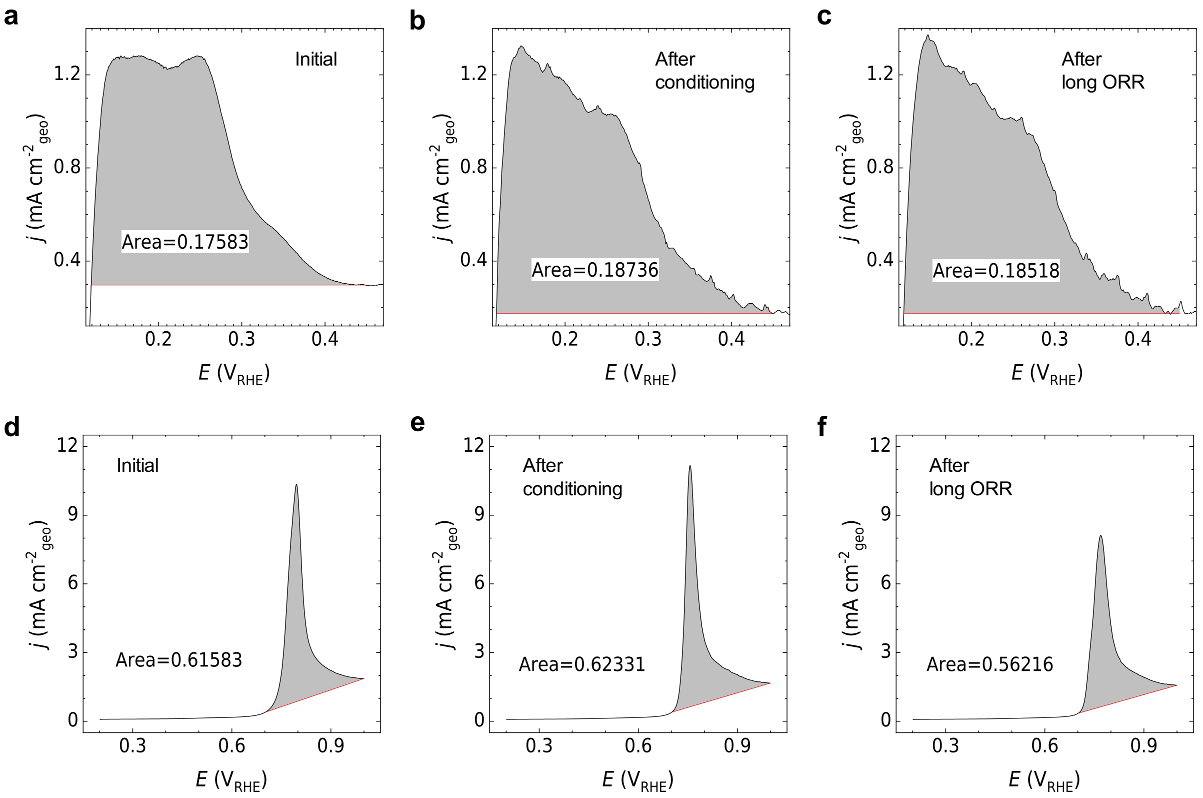

Supplementary Figure 12 | Determination of the change in surface area by hydrogen underpotential deposition (H-UPD) and CO displacement. a, H-UPD voltammograms of a reference Pt/c electrode (0.3 mg cm^{-2}) in the MEA after assembly, **b,** after performing the conditioning outlined in Supplementary Figure 4 and **c,** after 4h of continuous ORR at -800 mA cm^{-2}, 45 °C and pO_2 of 6 bars; followed by 100 cycles between 0.6-1 V_{RHE} at 5 mV s^{-1} and identical T and pO_2. The H-UPD surface area changes only by 5-6% after the conditioning and steady state ORR. **d-f,** CO stripping performed on the reference sample at the respective stages of panels **a-c**. The CO stripping charge shows a difference of up to 10% between panels e-f. An increase of 5-10% in the H-UPD and CO charge compared to the initial stage is consistent with the electron microscopy results in Supplementary Figure 6-11 that indicate varying mean particle sizes of up to 10% in the histograms. Importantly, these small changes in the surface area are consistent with the very high linear regression values reaching 0.999 in the Arrhenius analysis, which indicate negligible temperature dependent changes of the nanoparticle sizes between subsequent runs.

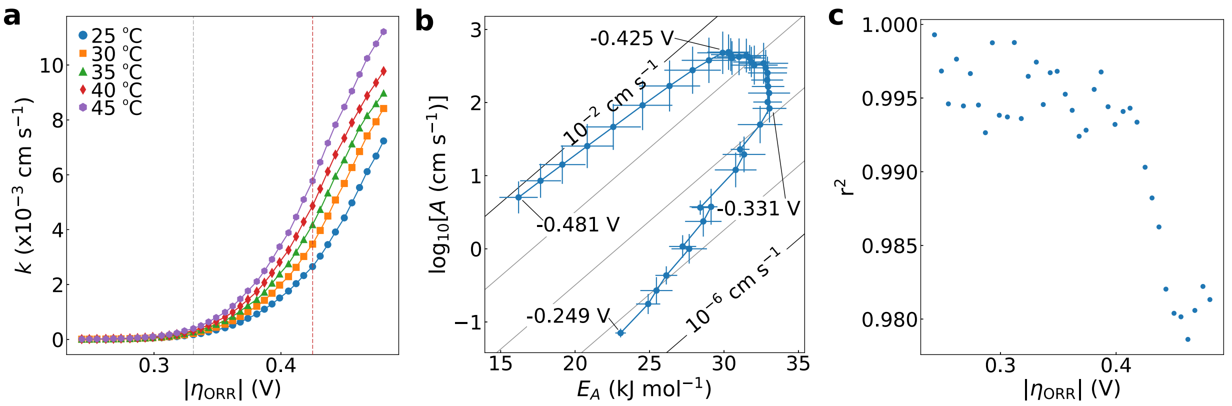

Supplementary Figure 13 | The impact of mass transport at higher overpotentials on the kinetic maps. **a,** Temperature dependent rate constants over whole overpotential range. For derivation, see Figure 2 and text in main, Supplementary Figure 22 and Supplementary Note 3. **b,** Kinetic map extracted from panel a for the whole overpotential range. At overpotentials > 0.425V, the mass transport limitations because clearly evident by a strong suppression of the pre-exponential factor. **c,** Despite the impact of mass transport, the r^2 linear regression from the Arrhenius analysis remain relatively high. This shows that high linear R^2 values alone do not rule out an impact of mass transport on the kinetics. All data for 190 μg cm^{-2} Pt/C.

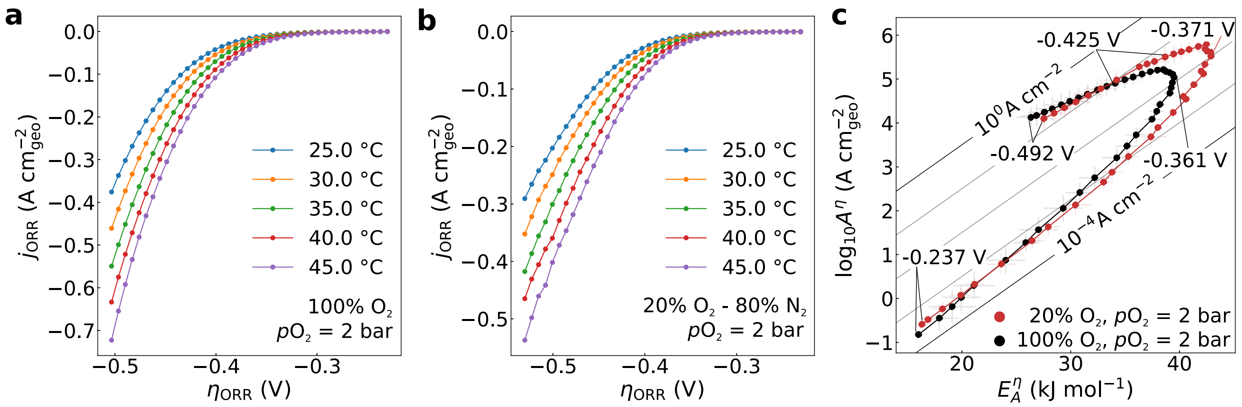

Supplementary Figure 14 | The impact of a reduced O_2 concentration in the gas feed on the kinetic maps. **a,** Temperature dependent polarization curves with 100% O_2 feed at $pO_2 = 2$ bar. **b,** Temperature dependent polarization curves of the same cell as in panel a, but with 20% O_2 feed at $pO_2 = 2$ bar, showing substantially suppressed rates. **c,** Overpotential dependent kinetic map with different O_2 feeds. At low overpotentials, the compensation region is almost identical and a turning point is also apparent for both curves. However, for the curve with reduced O_2 concentration, the overpotential of the turning point increases and the pre-exponential factor is suppressed at higher currents, which can explain why previous studies in liquid electrolytes with limited O_2 solubility did not observe the turning potential.

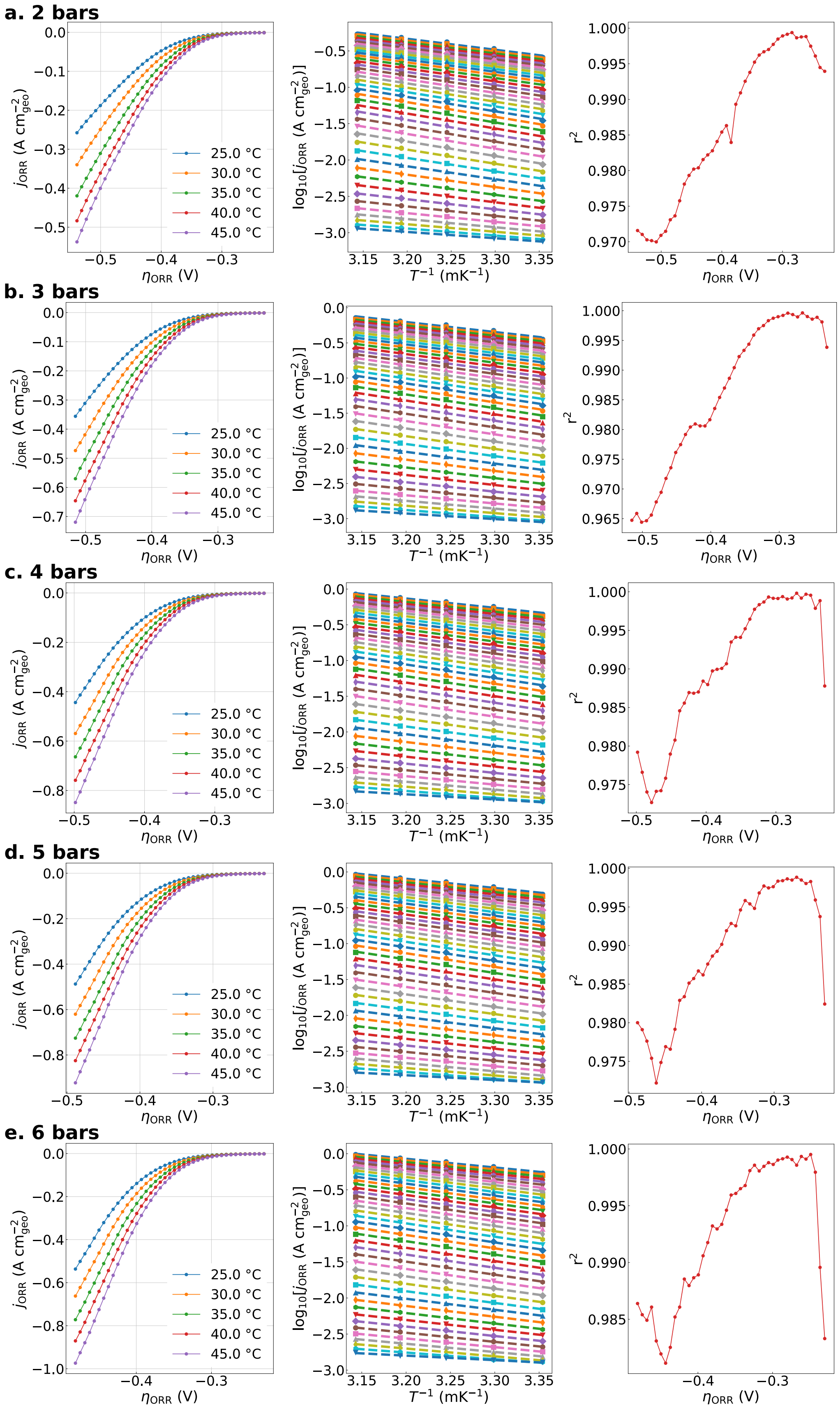

Supplementary Figure 15 | Arrhenius analysis of the ORR on Pt. a-e, Temperature dependent I-V curves, Arrhenius curves and linear regression R^2 values for the ORR on Pt/C between 2-6 bar, respectively. The overpotentials are corrected for the temperature and pressure dependence of the equilibrium potential, the *IR* drop and the HOR overpotential. The currents are corrected for the H_2 cross-over HOR current.

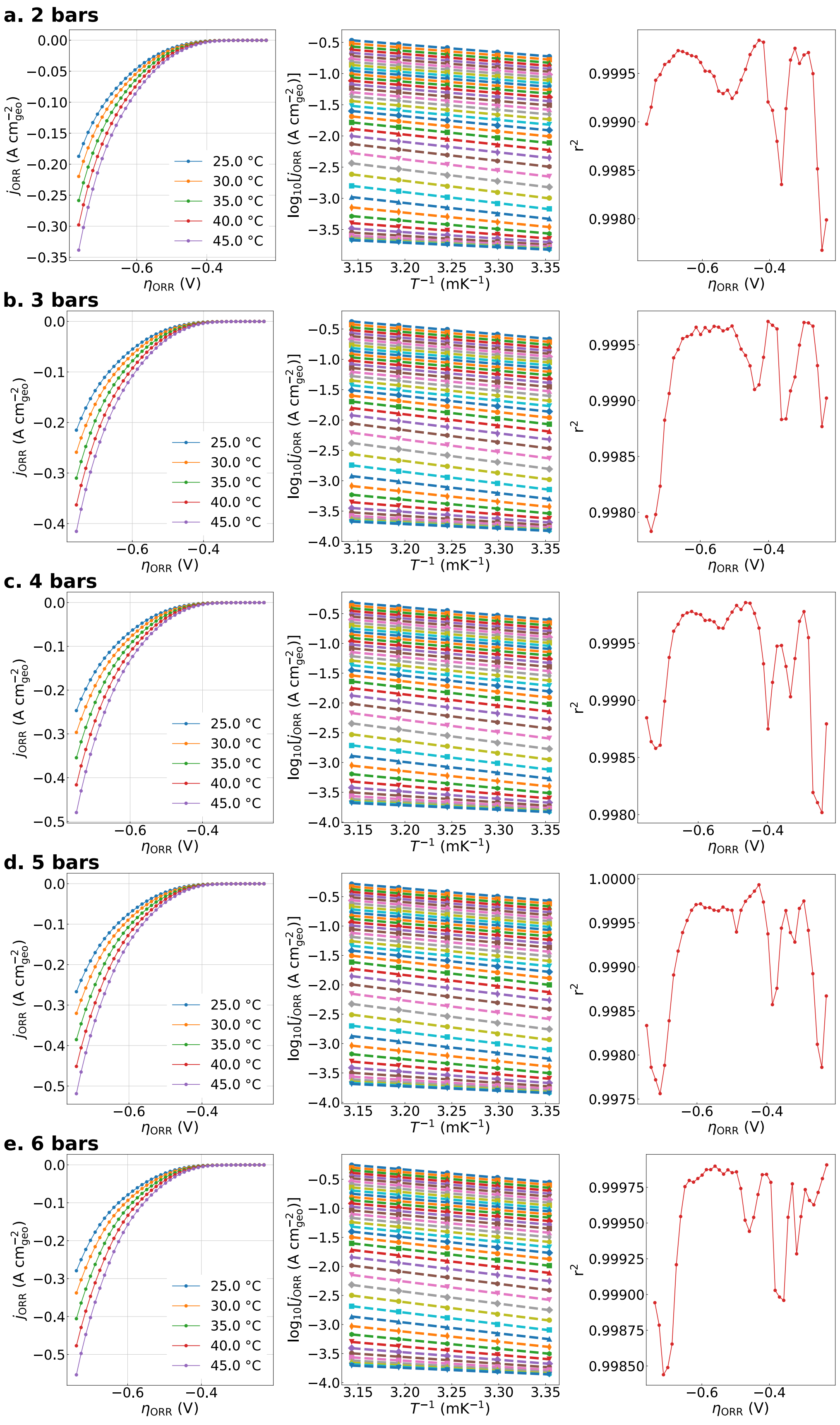

Supplementary Figure 16 | Arrhenius analysis of the ORR on Ir. a-e, Temperature dependent I-V curves, Arrhenius curves and linear regression R^2 values for the ORR on Ir/C between 2-6 bar, respectively. The overpotentials are corrected for the temperature and pressure dependence of the equilibrium potential, the *IR* drop and the HOR overpotential. The currents are corrected for the H_2 cross-over HOR current

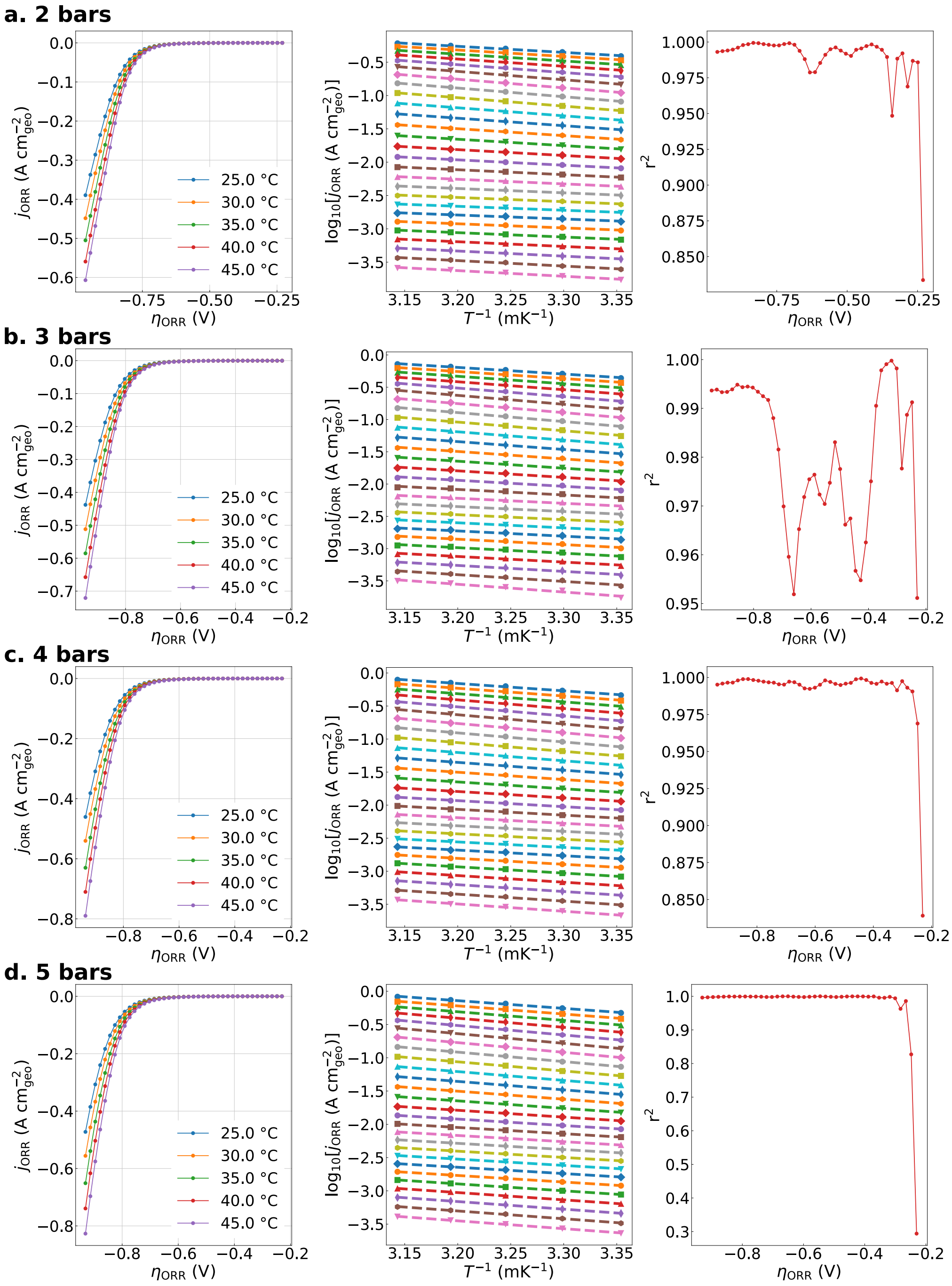

Supplementary Figure 17 | Arrhenius analysis of the ORR on Ru. a-e, Temperature dependent I-V curves, Arrhenius curves and linear regression R^2 values for the ORR on Ru/C between 2-6 bar, respectively. The overpotentials are corrected for the temperature and pressure dependence of the equilibrium potential, the *IR* drop and the HOR overpotential. The currents are corrected for the H_2 cross-over HOR current

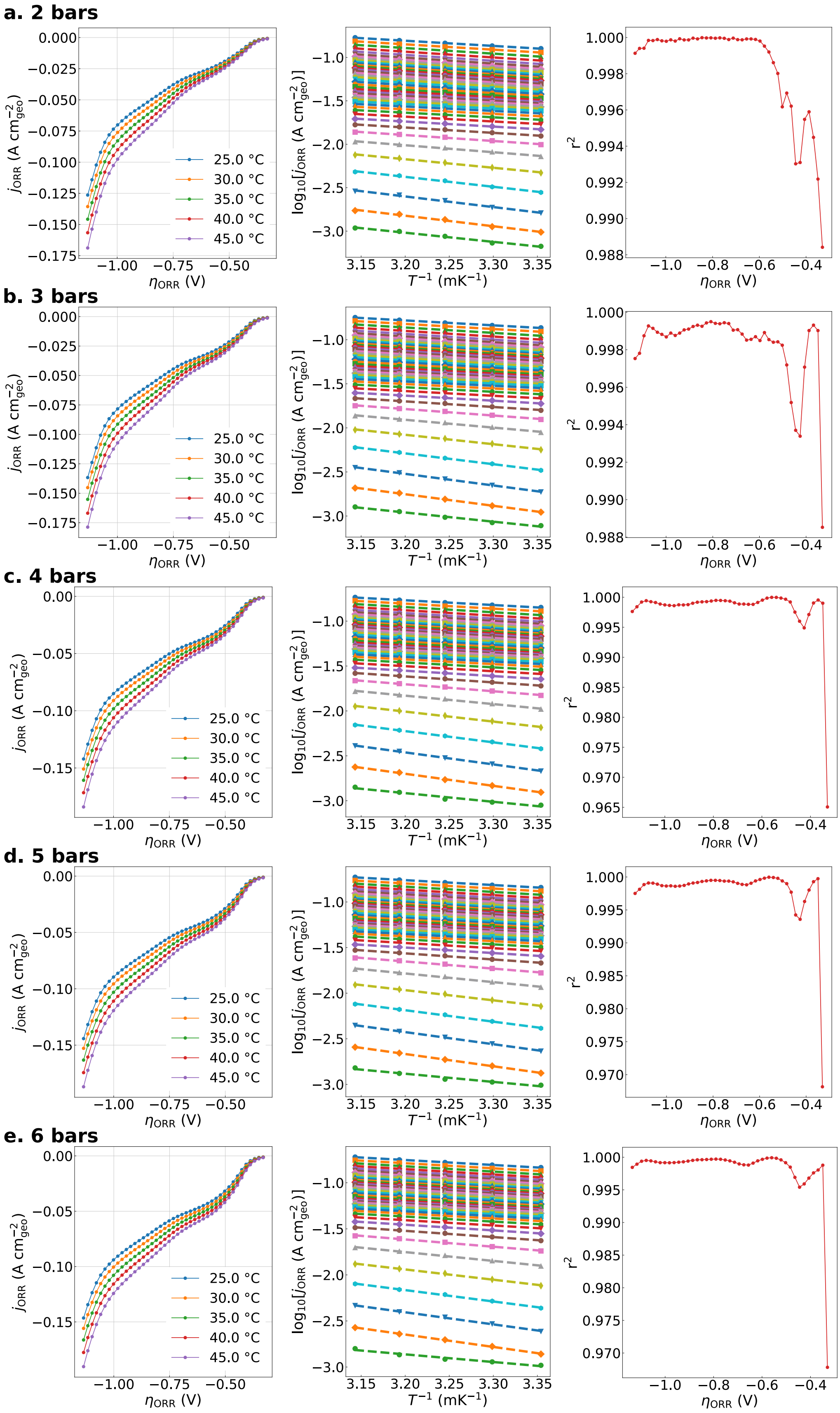

Supplementary Figure 18 | Arrhenius analysis of the ORR on Rh. a-e, Temperature dependent I-V curves, Arrhenius curves and linear regression R^2 values for the ORR on Rh/C between 2-6 bar, respectively. The overpotentials are corrected for the temperature and pressure dependence of the equilibrium potential, the *IR* drop and the HOR overpotential. The currents are corrected for the H_2 cross-over HOR current

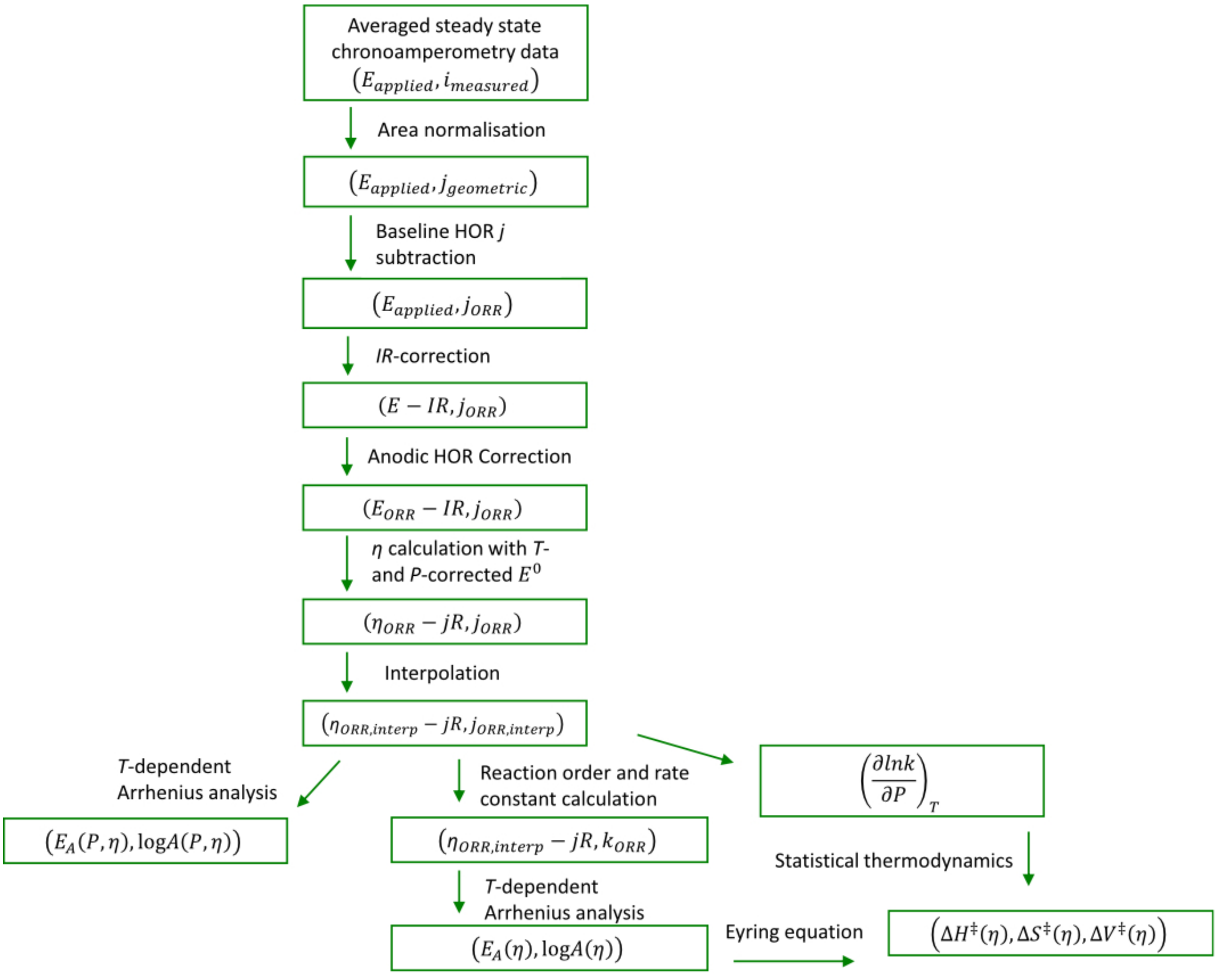

Supplementary Figure 19 | Analysis chart. Temperature- and pressure-dependent multi-step chronoamperometry data was analyzed to obtained the overpotential-dependent $\Delta H^{\ddagger}$, $\Delta S^{\ddagger}$ and $\Delta V^{\ddagger}$ for the ORR on Pt and Rh nanoparticles.

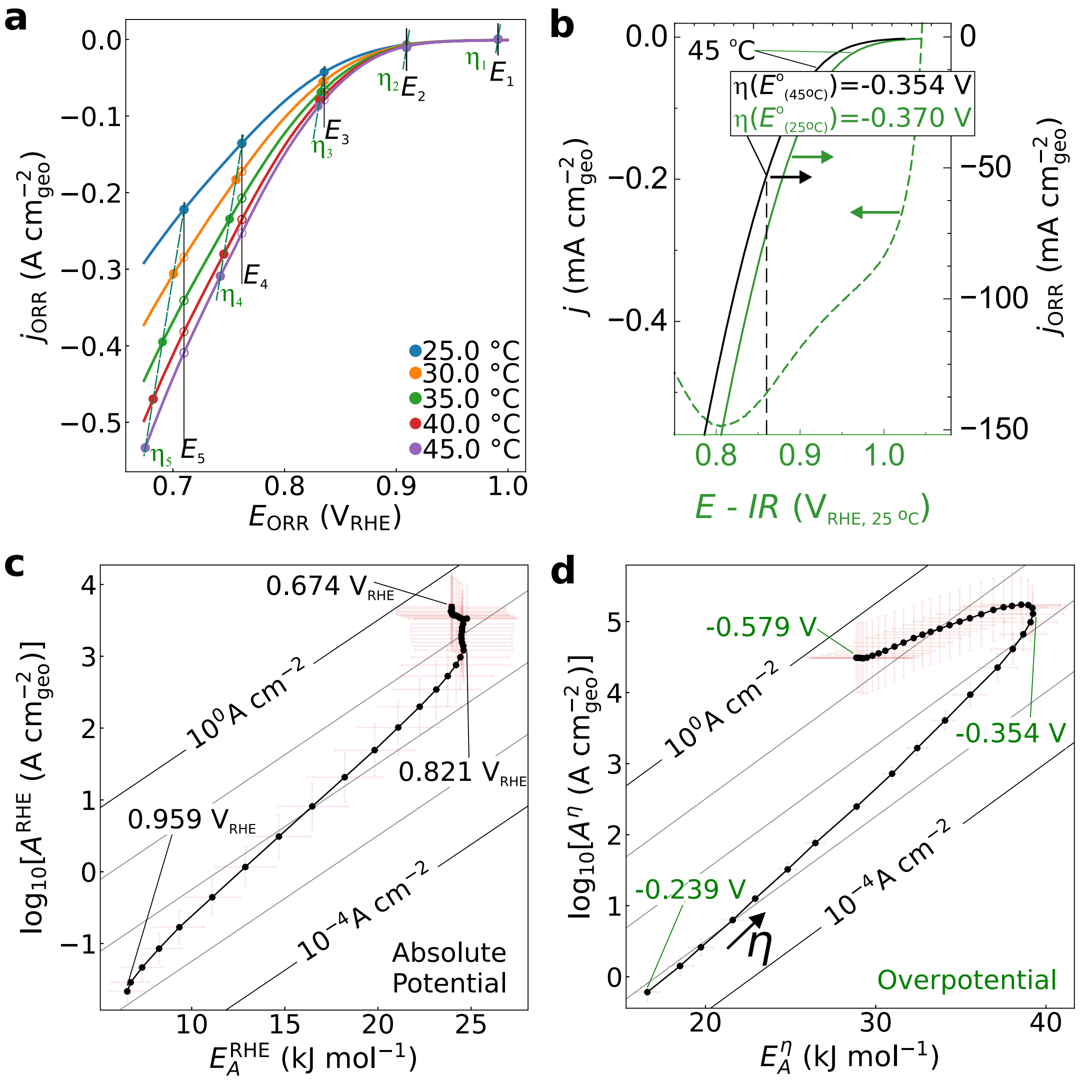

Supplementary Figure 20 | Kinetic maps with ideal and formal activation parameters. a, As measured ORR polarization curves on Pt/C at different temperatures and pO_2 = 2 bar on the RHE scale (void circles) and on overpotential scale (filled circles), considering the temperature dependent equilibrium potential, E^0. At a constant absolute potential, the applied overpotential decreases with increasing temperature, since E^0 decreases, too. **b,** Polarization curve of ORR measured at 45 °C and taking temperature corrected E^0 (black curve, y-axis to the right) and taking E^0 at 25˚C (green curve, y-axis to the right) on the RHE scale. The reduction peak of the voltammogram of Pt nanoparticles is shown and referenced on the RHE scale (dashed line in green, y-axis to the left). **c,** Kinetic map with formal activation parameters ($\log_{10} A^{RHE}$, E_A^{RHE}) derived from Arrhenius analysis as function of absolute potential, E_{RHE}. **d,** Kinetic map with real activation parameters ($\log_{10} A^{\eta}$, E_A^{η}) derived from Arrhenius analysis as function of overpotential, η, accounting for temperature dependence of the equilibrium potential. For details, see Methods.

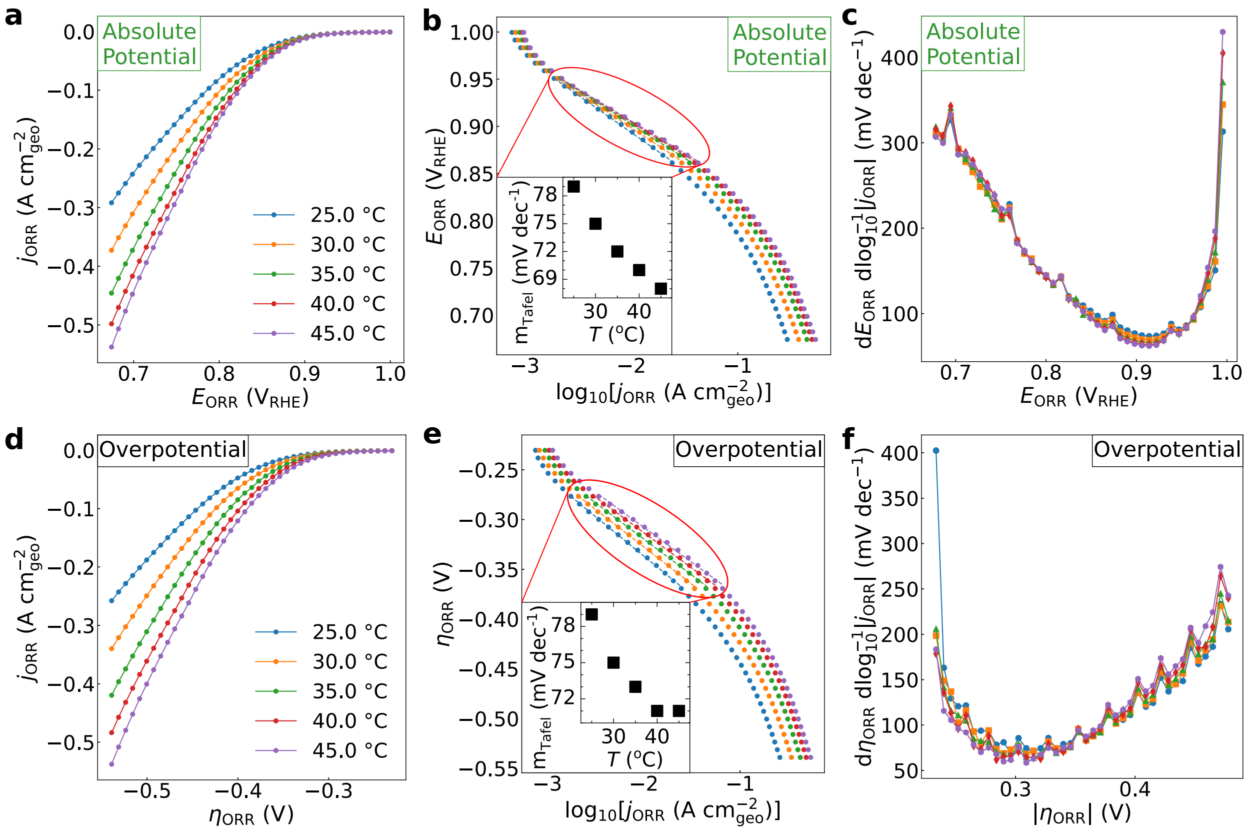

Supplementary Figure 21 | Impact of the temperature correction of the equilibrium potential on Tafel slopes. a-c, ORR polarization curves, Tafel plots and potential dependent Tafel slopes at different temperatures on absolute potential scale, E_{RHE}, respectively. **d-f,** ORR polarization curves, Tafel plots and potential dependent Tafel slopes at different temperatures on overpotential scale. Note, the region of (seemingly) linear Tafel slopes in b and e coincides with the lowest potential dependent Tafel slopes in c and f. This region represents a part of the compensation region, where rates increase due to an overcompensating Arrhenius pre-exponential factor (Supplementary Figure 16). However, despite the changing Tafel slopes beyond $\eta = 0.3V$, the kinetics are not strongly dominated by mass transport up to $\sim \eta = 0.425V$ as is evident in Supplementary Figure 18. All data for 190 µg cm^{-2} Pt/C.

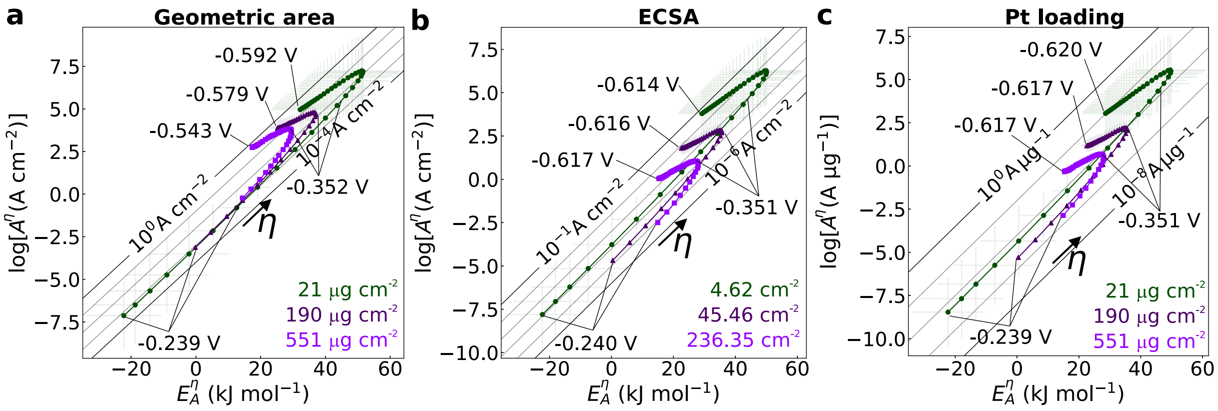

Supplementary Figure 22 | Impact of loading on the critical transition region of the ORR on Pt/C. Full range of cell overpotential dependent kinetic maps for ORR on Pt/C at pO_2 = 2 bar normalized by **a**, geometric area; **b,** electrochemical area calculated from H-UPD region and **c**, Pt loading per unit area. Strikingly, irrespective of the normalization procedure, the cell overpotential of maximum activation energy is essentially identical for the intermediate and higher loading (190-551 μg cm^{-2}). This strongly supports our view that the turning potential is informing on the intrinsic catalyst activity. Only at lower loading do we observe that a higher cell overpotential is needed to overcome the compensation zone. This might be caused by lateral mass transport to sparse sites in the GDE, but also the higher uncertainty for the lower loading (compare error bars between loadings). The cell overpotentials are corrected for the temperature and pressure dependence of the equilibrium potential. The currents are corrected for the H_2 cross-over HOR current. For better traceability, the cell overpotentials are uncorrected for the HOR overpotential. For the impact of the HOR overpotential in the kinetic maps, see Supplementary Figure 1-2.

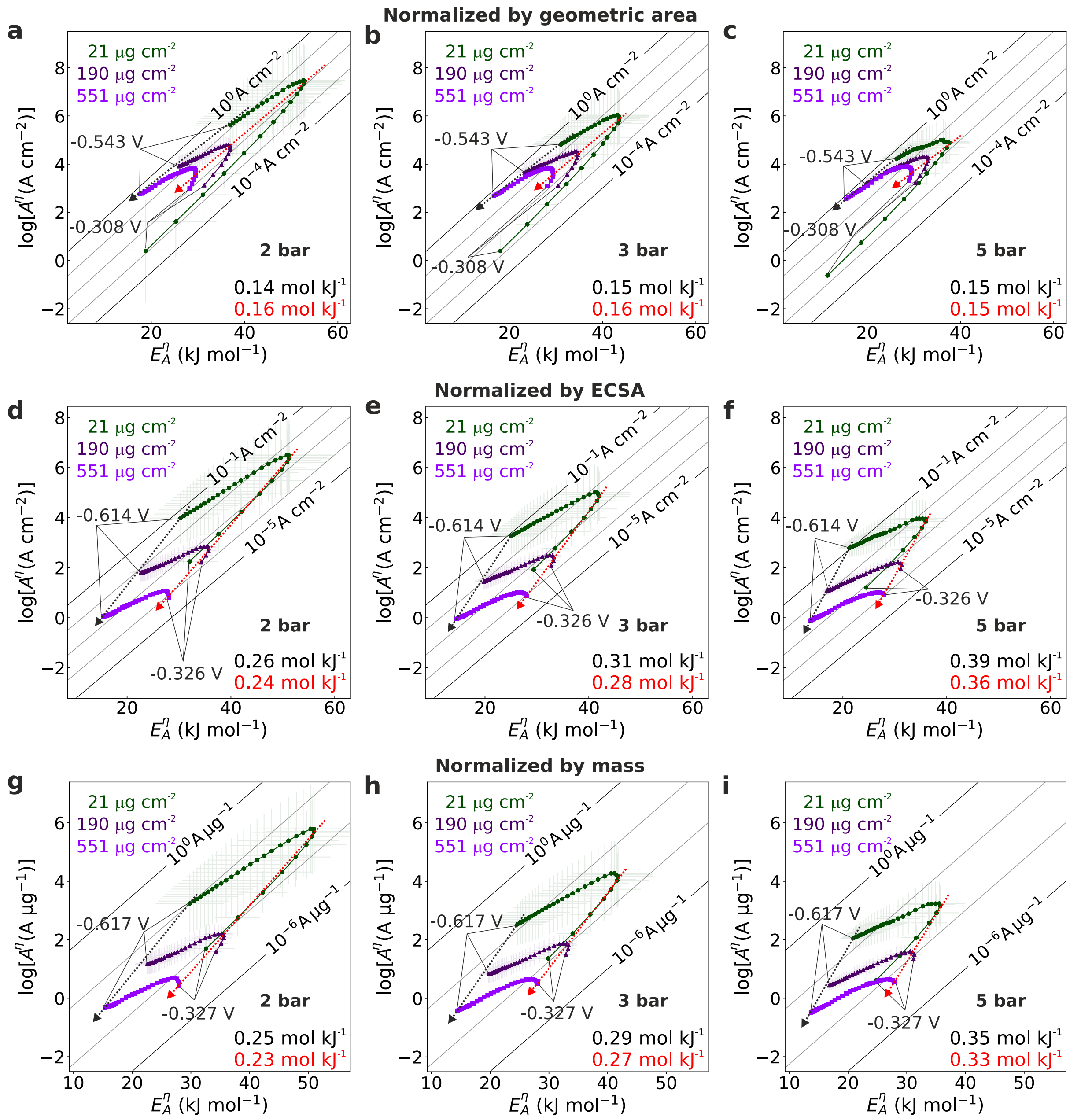

Supplementary Figure 23 | Variation of the kinetic maps for the ORR on Pt/C at different Pt loadings. Impact of the loading in the kinetic maps of the ORR on Pt/C obtained at different oxygen pressures normalized by **a-c,** geometrical area; **d-f,** specific area calculated from the H-UPD region (Supplementary Figure 23) and **g-i,** Pt mass loading. The slopes in red and black show the trend that is followed by E_A and $\log_{10} A$ at the transition point and at the maximum overpotential, respectively. The panels in a-c show very similar slopes for different pressures whereas, **d-f** and **g-i** show a higher slope with increasing pressure, indicative of a higher impact of the oxygen pressure for a low number of active sites. The cell overpotentials are corrected for the temperature and pressure dependence of the equilibrium potential. The currents are corrected for the H_2 cross-over HOR current. For better traceability, the cell overpotentials are uncorrected for the HOR overpotential. For the impact of the HOR overpotential in the kinetic maps, see Supplementary Figure 1-2.

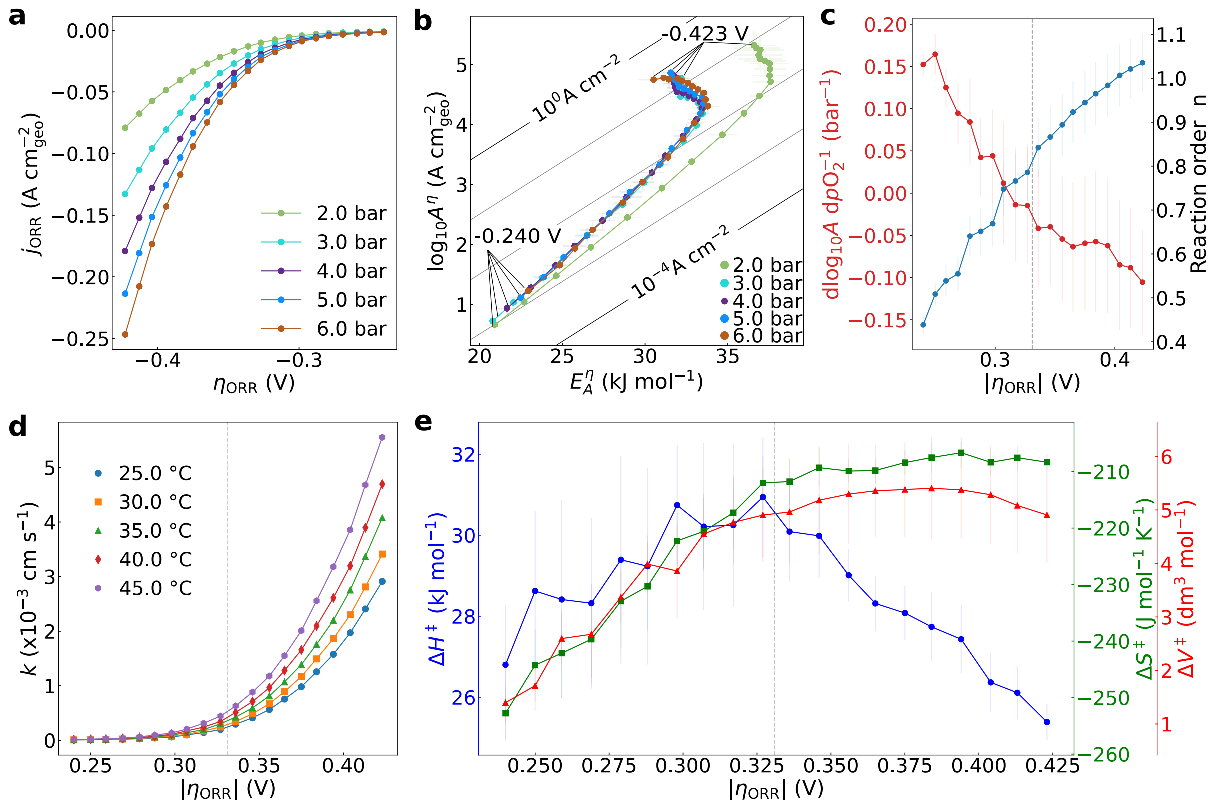

Supplementary Figure 24 | Additional dataset for the oxygen pressure dependence on the ORR kinetics. **a,** ORR polarization curves at 25°C and different oxygen pressures (pO_2). **b,** Kinetic maps ($\log_{10} A^{\mathrm{RHE}}$ *vs.* E_A^{RHE}) for the different pressures calculated from temperature dependent rates as function of overpotential, η. **c,** Overpotential dependent reaction order and changes in the Arrhenius pre-exponential factor with O_2 pressure, $\mathrm{dlog}_{10}\,\mathrm{A}\;\mathrm{d}pO_2^{-1}$. Clearly, the changes in the pre-exponential factor cannot solely originate from the positive and increasing reaction order. **d,** overpotential dependent ORR rate constant, after accounting for reaction order. **e,** Overpotential dependent activation enthalpy, $\Delta H^{\ddagger}$, activation entropy, $\Delta S^{\ddagger}$, and activation volume, $\Delta V^{\ddagger}$. Error bars are based on linear regression R^2 values. For all measurements a Pt mass-loading of 190 µg cm^{-2} was used. This second dataset shows the same trends as those shown in Figure 1-3, although a slightly different potential window was explored and coarse potential jumps were performed, proving the robustness of the obtained results.

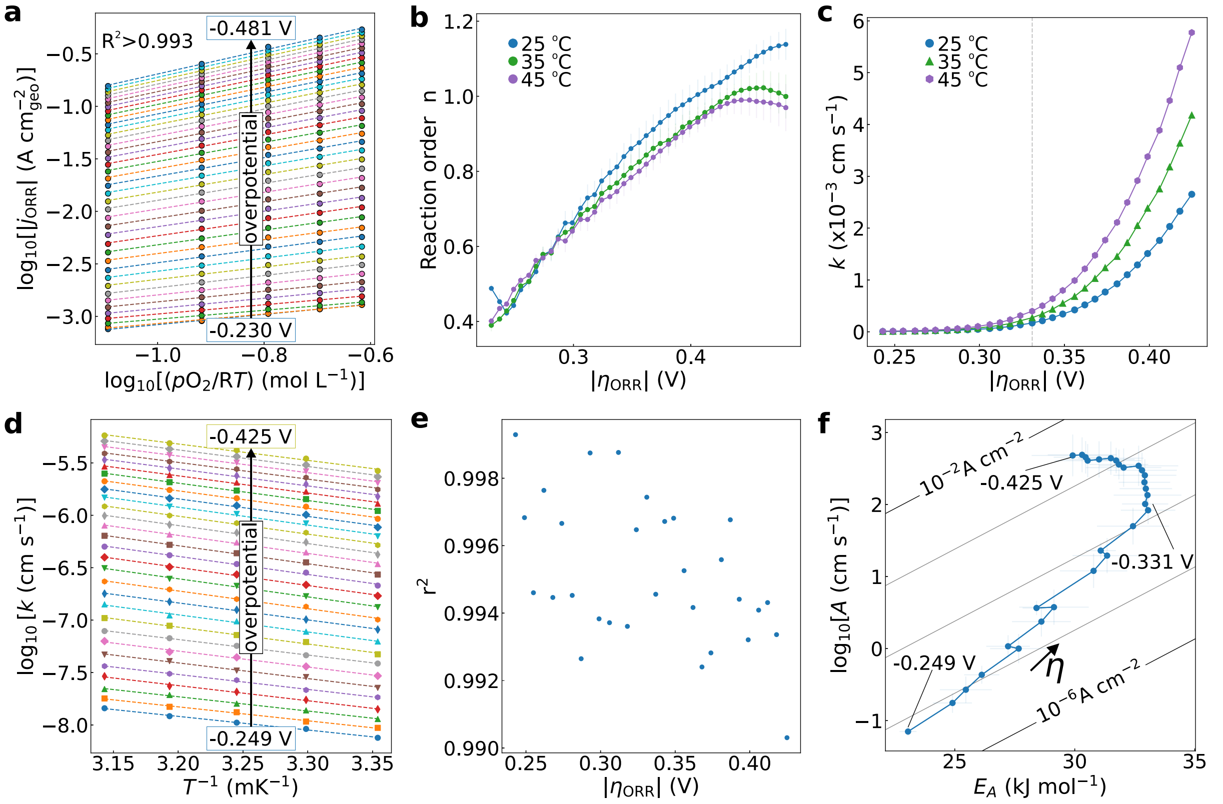

Supplementary Figure 25 | Determination of the reaction order and reaction rate constant from pressure dependent currents. a, Determination of the oxygen reaction order and rate constant from the logarithm of the ORR current density over the logarithm of oxygen pressure at various overpotentials at 25 °C, as described by Equation 12 in Supplementary Note 4. Same analysis was performed for all temperatures (not shown). **b,** Reaction order of oxygen, extracted from the slope of the linear fits in panel a, shown as function of the overpotential for different temperatures. **c,** Reaction rate constant (*k*) obtained from the intercepts of the linear fits in panel a as function of the overpotential at different temperatures. **d,** Arrhenius plots of the reaction rate constant in panel c at different overpotentials. **e,** Linear regression values (R^2) of panel d, supporting the quality of the Arrhenius fits across the overpotential range. **f,** Kinetic map generated from the Arrhenius analysis in panel d, highlighting the close similarity to the kinetic maps based on Arrhenius analysis of temperature dependent currents in the main manuscript. Note, here, the pressure dependence of the current density is used to separate the reaction order from the rate constant. The temperature dependence of the rate constant is then used to extract the kinetic map. As a result, we only obtain a "general", pressure independent kinetic map.

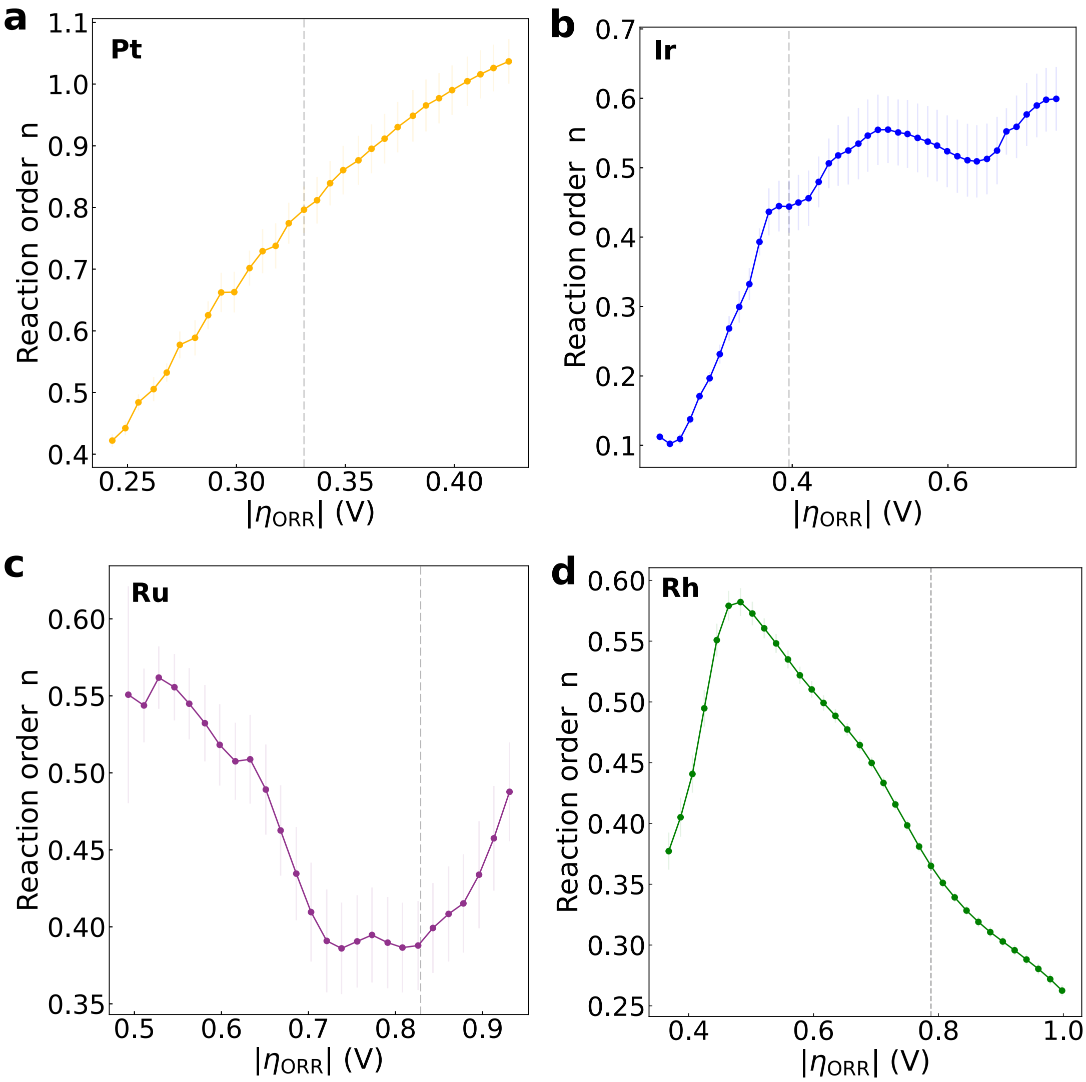

Supplementary Figure 26 | Overpotential dependent reaction orders for different catalysts. Variation of the ORR reaction order with the overpotential for Pt/C (190 µg cm^{-2}), **b,** Ir/C (250 µg cm^{-2}), **c,** Ru/C (180 µg cm^{-2}) and, **d, Rh/C** (190 µg cm^{-2}). The methodology for determining the reaction order is described in Supplementary Note 4, and Pt/C is shown as an example in Supplementary Figure 25.

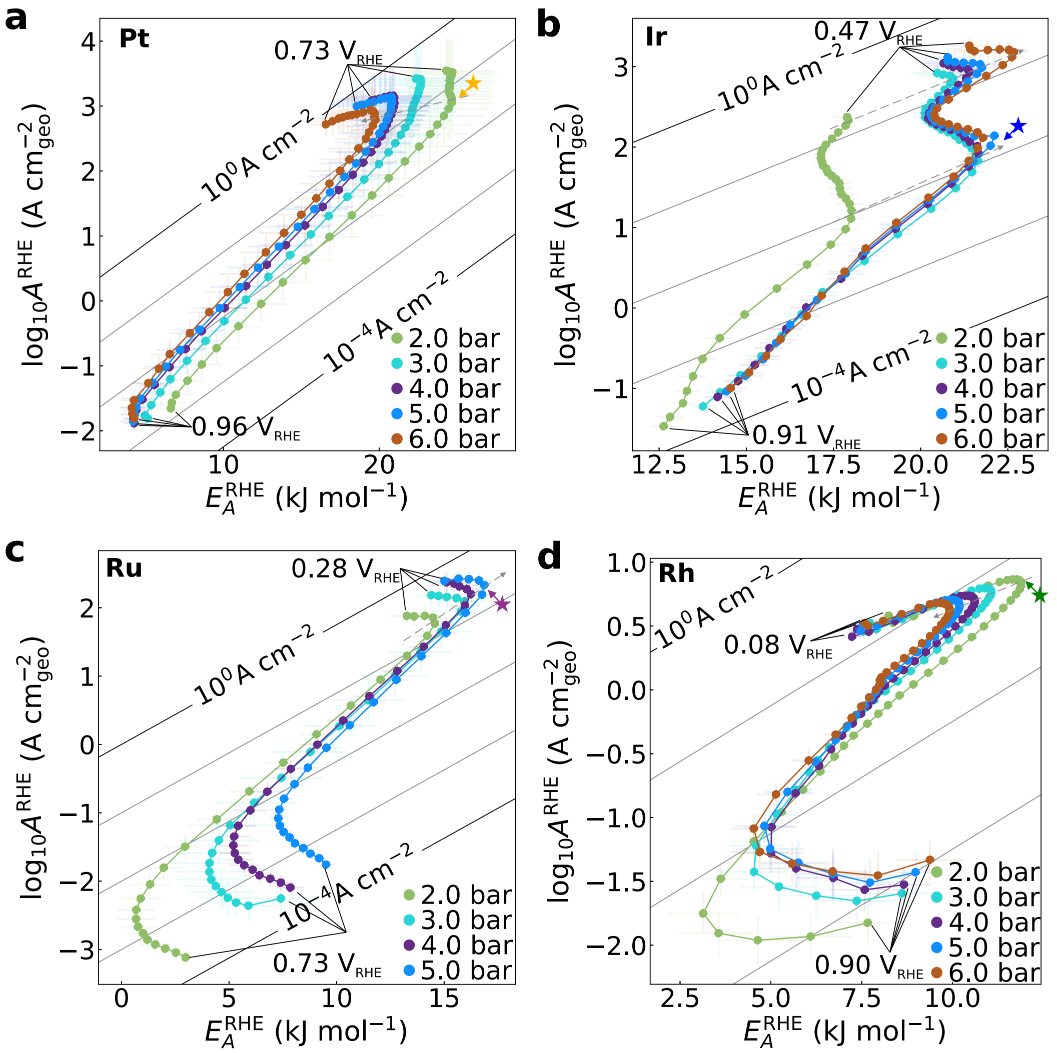

Supplementary Figure 27 | Kinetic map with formal activation parameters ($\log_{10} A^{RHE}$, E_A^{RHE}) derived from Arrhenius analysis as function of absolute potential, E_{RHE}. a, Kinetic map for Pt/C (190 µg cm^{-2}), **b,** Ir/C (250 µg cm^{-2}), **c,** Ru/C (180 µg cm^{-2}) and, **d, Rh/C** (190 µg cm^{-2}). For the difference between the formal and real activation parameters, see Supplementary Note 3 and Supplementary Figure 20.

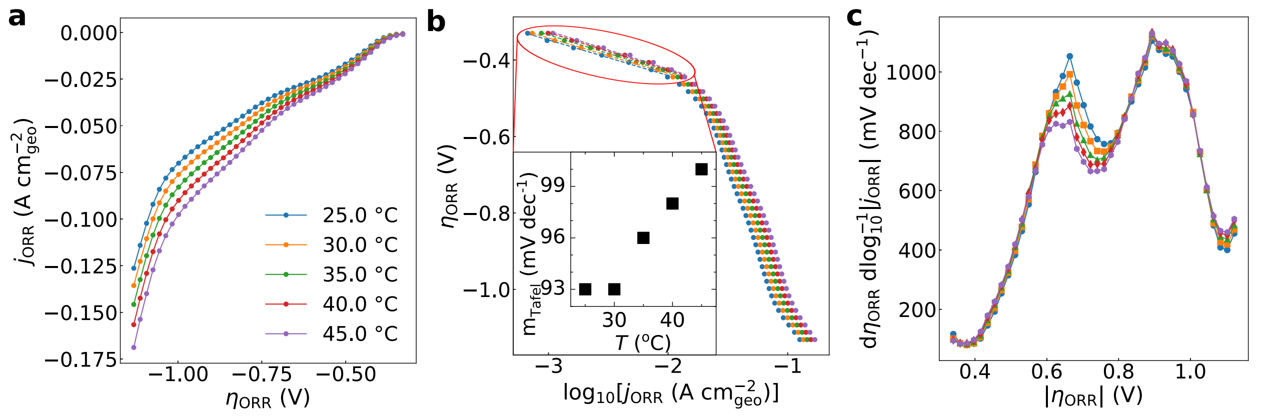

Supplementary Figure 28 | Overpotential dependent Tafel slopes for Rh/C. a, Steady-state polarization curves of oxygen reduction reaction at different temperatures and 2 bar O_2. **b,** Tafel plot with short linear regime at lower overpotentials. **c,** Bias dependent Tafel slope extracted from panel b. The Tafel slopes reach very high values, which are typically associated with mass transport limitations in the literature[19,20]. However, the current densities for Rh/C (≤ 150mA cm^{-2}) are well below the ones that allow us to study ORR kinetics on Pt/C with the MEA (≤ 250mA cm^{-2}). The kinetic maps Fig. 4 show rich bias dependent entropic and enthalpic changes. Thus, non-linear and strongly increasing Tafel slopes as a function of bias are not necessarily caused by mass-transport limitations. The analysis of constant Tafel slopes at a constant temperature rests on the assumption that the reaction is enthalpically driven with constant, purely enthalpic charge transfer coefficients. In all generality, this is invalid for inner-sphere reactions, which can possess strongly bias dependent enthalpic and entropic components that can even (partially) compensate. This can lead to very high bias dependent Tafel slopes, that are rooted in the rich kinetics and unrelated to mass transport limitations. However, to reliably analyze this, cells with high mass transport are needed. For liquid electrolyte cells with limited mass transport, high Tafel slopes should indeed be carefully considered before attempting to extract kinetic information at higher current densities[19,20]. For a new reaction or conditions, we recommend to always perform temperature dependent kinetic studies first to assess whether the reaction is indeed enthalpically driven and whether linear Tafel slopes at a constant temperature inform on the kinetics.

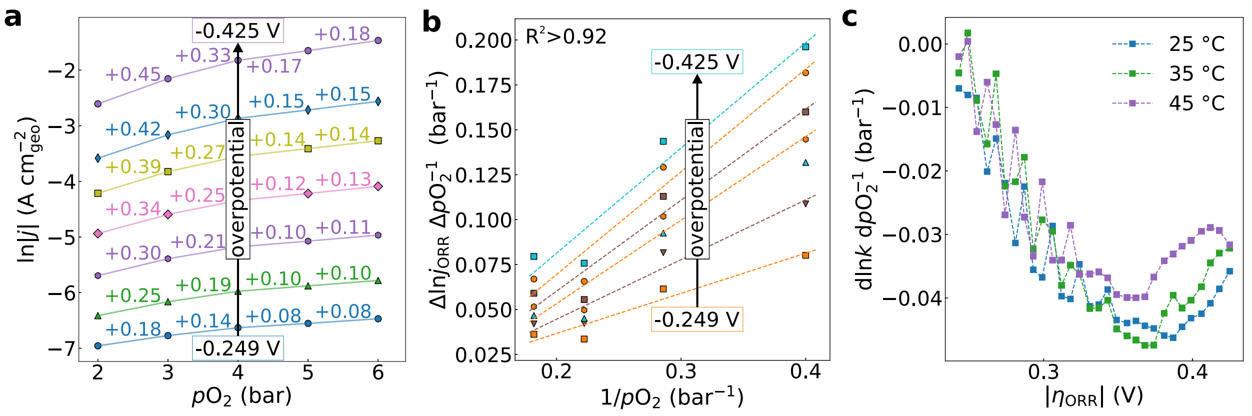

Supplementary Figure 29| Impact of oxygen pressure variation on the reaction rate constant. Based on Equation 13 in Supplementary Note 4, changes in oxygen pressure influence the reaction rate. **a,** Natural logarithm of the ORR current density ($\ln|j_{ORR}|$) plotted against oxygen pressure at various overpotentials at 25 °C. The numerical labels between data points indicate the slope ($\Delta\ln|j_{ORR}|/\Delta pO_2$) for each pressure interval, quantifying the sensitivity of the current to pressure. **b,** Variation of the calculated slopes from panel **a** plotted against the inverse of oxygen pressure ($1/pO_2$) for different overpotentials at 25 °C. According to Equation 7 in Supplementary Note 4, the slope of these linear fits yields the reaction order with respect to oxygen, while the interpolation to the y-intercept provides the pressure dependence of the rate constant ($d\ln k/dpO_2$). Note, this interpolation has a higher sensitivity to low pressure changes than to high pressure changes. **c,** Change of $d\ln k/dpO_2$ as function of the overpotential for different temperatures. $d\ln k/dpO_2$ can be used for the calculation of the activation volume as outlined in Supplementary Notes 4 and 5.

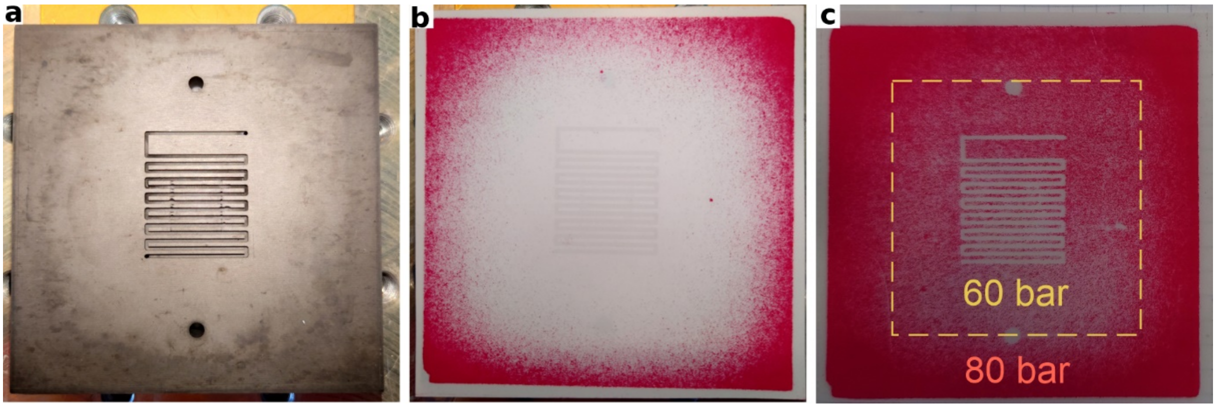

Supplementary Figure 30 | Pressure distribution inside the MEA cell. **a,** Graphite block with serpentine-patterned flow field. **b,** Pressure distribution print inside the MEA cell showing concentrated pressure along the edges due to perimeter screws. **c,** Pressure distribution print with the compression jacket, demonstrating improved uniformity compared to panel b. The MEA cell, constructed with 2.1 × 2.1 cm graphite blocks and pre-hydrated Nafion 212 membranes, was sealed with screws tightened to 4 Nm torque. The addition of a stainless-steel compression jacket, featuring a 3.0 × 3.0 cm raised central region, redistributed pressure more evenly, as confirmed by pressure-sensitive paper. This optimization reduced gas crossover between chambers by isolating guide pin holes and ensuring effective sealing, with approximately 60 bars pressure applied. Residual edge pressure disparities were observed but did not impact cell performance.

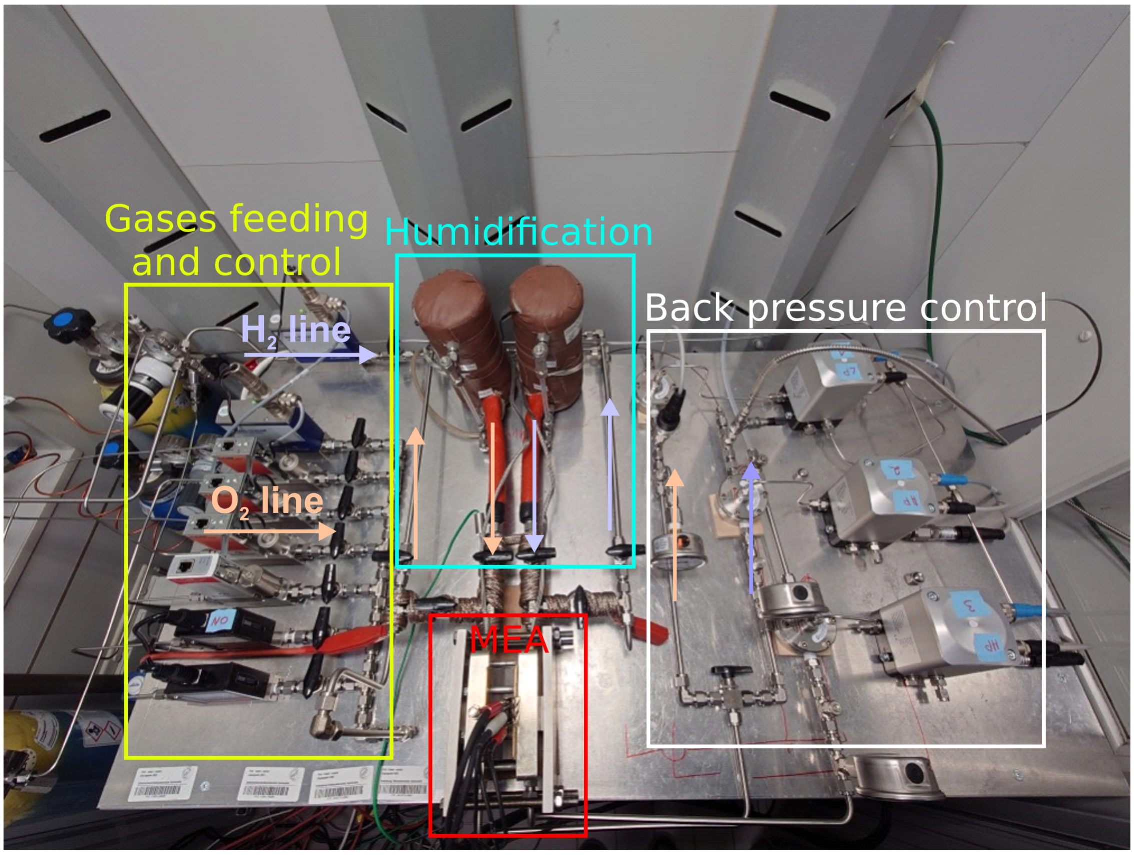

Supplementary Figure 31 | Experimental setup for pressure- and temperature-dependent measurements. Top-down view highlighting gas flow paths. Two independently regulated gas supply lines, each connected to humidification bottles for relative humidity control, were employed. One line exclusively supplied H_2, while the other used a manifold with multiple mass flow controllers (MFCs) for varied gas delivery. A gas mixer downstream ensured homogeneous gas composition when required. Pure O_2 was used for ORR experiments, and pure N_2 for blank measurements. Downstream of the MEA, back-pressure regulators coupled with electronic valves and pressure sensors in a negative feedback configuration maintained stable pressure, with argon providing 10 bar (gauge) for accentuation of the Ar pressure-controlled valves. The MEA (red square) was heated using embedded metallic bars in brass blocks, with temperatures electronically controlled *via* a K-type thermocouple and a custom-built Eurotherm 3508-based controller (not shown). The MEA is additionally surrounded with a stainless-steel compression jacket that exerts mechanical pressure in the center of the square-shaped MEA (Supplementary Figure 25). The system also controlled the temperatures of the humidification bottles (Fuel Cell Technologies), outlet lines, and bypass pathway.

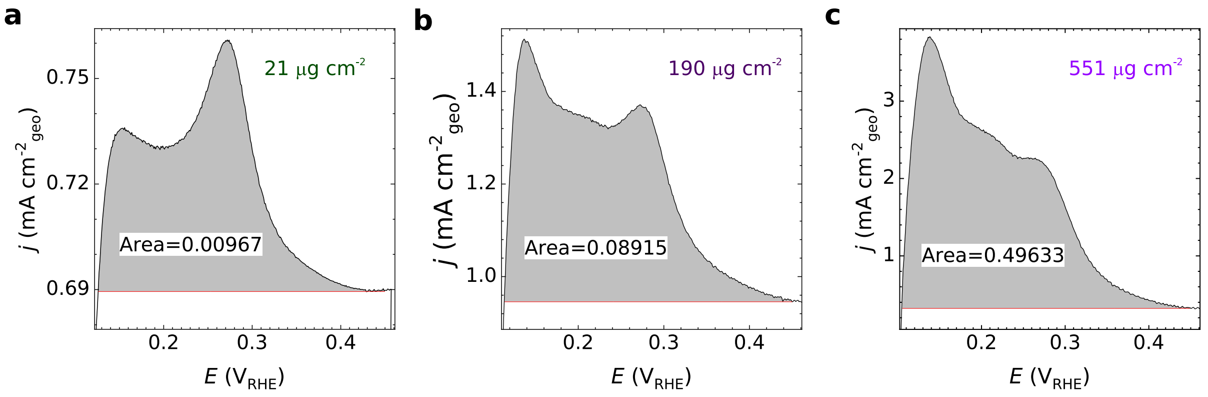

Supplementary Figure 32 | Determination of the specific area of the Pt nanoparticles at different loadings. a-c, H-UPD zone of the voltammograms of different Pt loadings at 25 ˚C and 10 mV s^{-1} for the determination of the specific area by integration of the H-UPD zone. The specific charge for the calculation of the area was 0.21 mC cm^{-2}.

Dispersion	Metal loading (wt %)	Carbon Support	Source	Particle Size (nm)	Metal surface area ($m^2 g^{-1}$)
**Pt/C**	70	Ketjenblack EC-300J	Fuel Cell Store	3-4	70
**Ir/C**	40	Vulcan XC-72	Fuel Cell Store	4-6	60
**Ru/C**	40	Ketjenblack EC-300J	Fuel Cell Store	4-7	200
**Rh/C**	20	Vulcan XC-72	Fuel Cell Store	2-4	120
**Pt black**	100	-	Fuel Cell Store	5-7.5	45-52

Supplementary Table 1. Properties of the starting materials used for electrode preparation. Commercial dispersions of nanoparticles on carbon were used to fabricate cathodes for the ORR, while Pt black was employed to prepare the anodes for the HOR. All nanoparticle dispersions were used as received, shortly after opening. The flasks were stored sealed and wrapped with Parafilm to minimize contamination during storage in the cabinet. After each use, the flasks were closed under ambient atmospheric conditions to maintain material integrity.

Dispersion	Dispersion mass (mg)	Nafion ionomer solution 5% (mg)	Water (g)	Isopropanol (g)	Total loading (mg cm^{-2})	Metal loading (mg cm^{-2})
**Pt/C**	4.02	24.04	0.52583	1.83961	0.04	0.02
**Pt/C**	20.33	79.10	0.52586	1.70924	0.33	0.19
**Pt/C**	80.10	332.3	0.51440	0.70531	0.95	0.55
**Ir/C**	70.50	360.61	0.51732	1.76991	0.78	0.25
**Ru/C**	46.88	246.64	0.50350	1.70462	0.56	0.18
**Rh/C**	69.82	77.97	0.50196	1.69559	0.99	0.19
**Pt black**	152.07	762.19	0.51194	1.70697	2.62	2.09

Supplementary Table 2. Preparation details of inks spray-coated onto GDL electrodes. Inks were prepared by weighing components into 10 mL borosilicate vials and homogenized via sonication using a horn sonotrode in an ice bath to prevent heating. Standard sonication was performed in 5-second pulses with 3-second pauses for 15 minutes at 60% power, while Pt black-based inks required 45 minutes at 70% power due to higher Pt loading. The inks were manually spray-coated onto 5.3 × 5.3 cm Freudenberg H23C2 porous carbon papers heated to 75 °C, cooled to room temperature, and weighed to determine total loading. Final electrodes (1.0 × 1.0 cm) were cut with precision tools to ensure consistency.

Supplementary References

1. Lazaridis, T., Stühmeier, B. M., Gasteiger, H. A. & El-Sayed, H. A. Capabilities and limitations of rotating disk electrodes versus membrane electrode assemblies in the investigation of electrocatalysts. *Nat Catal* **5**, 363–373 (2022).
2. Durst, J., Simon, C., Hasché, F. & Gasteiger, H. A. Hydrogen Oxidation and Evolution Reaction Kinetics on Carbon Supported Pt, Ir, Rh, and Pd Electrocatalysts in Acidic Media. *J. Electrochem. Soc.* **162**, F190–F203 (2015).
3. Sheng, W., Gasteiger, H. A. & Shao-Horn, Y. Hydrogen Oxidation and Evolution Reaction Kinetics on Platinum: Acid vs Alkaline Electrolytes. *J. Electrochem. Soc.* **157**, B1529 (2010).
4. Gisbert-González, J. M. *et al.* Bias Dependence of the Transition State of the Hydrogen Evolution Reaction. *J. Am. Chem. Soc.* (2025) doi:10.1021/jacs.4c18638.
5. Gómez-Marín, A. M., Rizo, R. & Feliu, J. M. Some reflections on the understanding of the oxygen reduction reaction at Pt(111). *Beilstein J. Nanotechnol.* **4**, 956–967 (2013).
6. Clouser, S. J., Huang, J. C. & Yeager, E. Temperature dependence of the Tafel slope for oxygen reduction on platinum in concentrated phosphoric acid. *J Appl Electrochem* **23**, 597–605 (1993).
7. Solorza-Feria, O. & DuroÃn, S. Temperature effects for oxygen reduction on Ru-nanoparticles in acid solution. *International Journal of Hydrogen Energy* **27**, 451–455 (2002).
8. Sarabia, F., Gomez Rodellar, C., Roldan Cuenya, B. & Oener, S. Z. Exploring dynamic solvation kinetics at electrocatalyst surfaces. *Nat Commun* **15**, 8204 (2024).
9. Petersen, H. A. *et al.* On the Temperature Sensitivity of Electrochemical Reaction Thermodynamics. *ACS Phys. Chem Au* **3**, 241–251 (2023).
10. Protsenko, V. S. & Danilov, F. I. Activation energy of electrochemical reaction measured at a constant value of electrode potential. *Journal of Electroanalytical Chemistry* **651**, 105–110 (2011).
11. Logan, S. R. The origin and status of the Arrhenius equation. *J. Chem. Educ.* **59**, 279 (1982).
12. Rowlinson *, J. S. The Maxwell–Boltzmann distribution. *Molecular Physics* **103**, 2821–2828 (2005).
13. Jeevanandam, P. & Vasudevan, S. Arrhenius and non-Arrhenius conductivities in intercalated polymer electrolytes. *The Journal of Chemical Physics* **109**, 8109–8117 (1998).
14. Chatgilialoglu, C., Ingold, K. U. & Scaiano, J. C. Rate constants and Arrhenius parameters for the reactions of primary, secondary, and tertiary alkyl radicals with tri-n-butyltin hydride. *J. Am. Chem. Soc.* **103**, 7739–7742 (1981).
15. Conway, B. E. & Wilkinson, D. F. Entropic and enthalpic components of the symmetry factor for electrochemical proton transfer from various proton donors over a wide temperature range. *Journal of Electroanalytical Chemistry and Interfacial Electrochemistry* **214**, 633–653 (1986).
16. Conway, B. E., Phillips, Y. & Qian, S. Y. Surface electrochemistry and kinetics of anodic bromine formation at platinum. *Faraday Trans.* **91**, 283 (1995).
17. Conway, B. E., Tessier, D. F. & Wilkinson, D. P. Experimental evidence for the potential-dependence of entropy of activation in electrochemical reactions in relations to the temperature-dependence of tafel slopes. *Journal of Electroanalytical Chemistry and Interfacial Electrochemistry* **199**, 249–269 (1986).
18. Muller, P. Glossary of terms used in physical organic chemistry (IUPAC Recommendations 1994). *Pure and Applied Chemistry* **66**, 1077–1184 (1994).
19. Van Der Heijden, O., Park, S., Vos, R. E., Eggebeen, J. J. J. & Koper, M. T. M. Tafel Slope Plot as a Tool to Analyze Electrocatalytic Reactions. *ACS Energy Lett.* 1871–1879 (2024) doi:10.1021/acsenergylett.4c00266.
20. Van Der Heijden, O., Park, S., Eggebeen, J. J. J. & Koper, M. T. M. Non-Kinetic Effects Convolute Activity and Tafel Analysis for the Alkaline Oxygen Evolution Reaction on NiFeOOH Electrocatalysts. *Angewandte Chemie* **135**, e202216477 (2023).
